# Ideal treatment timing of orthodontic anomalies—a German clinical S3 practice guideline

**DOI:** 10.1007/s00056-022-00409-3

**Published:** 2022-06-17

**Authors:** Christian Kirschneck, Peter Proff, Christopher Lux

**Affiliations:** 1 Department of Orthodontics, University Hospital Regensburg, Franz-Josef-Strauss-Allee 11, 93053 Regensburg, Germany; 2 Department of Orthodontics, University Hospital Heidelberg, Im Neuenheimer Feld 400, 69120 Heidelberg, Germany

**Keywords:** Malocclusion, Interceptive therapy, Dysgnathia, Dentition, Orofacial dyskinesia, Malokklusion, Interzeptive Behandlung, Dysgnathie, Dentition, Orofaziale Dyskinesie

## Abstract

**Purpose:**

Ideal treatment timing in orthodontics is controversially discussed depending on the type and extent of the dysgnathia and malocclusion present, especially with regard to efficiency, patient burden and treatment efforts of early compared to regular or late treatment. This German clinical practice guideline aims to clarify, at which time points an orthodontic anomaly can be effectively treated and how treatment efficiency differs depending on treatment timing.

**Methods:**

A systematic literature search was performed in various guideline databases and databases PROSPERO, MEDLINE (PubMed), Cochrane Library, Web of Science, ClinicalTrials.gov and the International Clinical Trials Registry Platform according to a predefined PICO (Population, Intervention, Comparison and Outcomes with added qualitative search terms) search algorithm and strategy. Appraisal of scientific evidence of the individual studies checked for eligibility was carried out according to SIGN (Scottish Intercollegiate Guidelines Network), AMSTAR II (Assessing the Methodological Quality of Systemic Reviews), and AXIS (Appraisal Tool to Assess the Quality of Cross-sectional Studies) tools. Only controlled studies with a high, acceptable or moderate quality (and thus an acceptable risk of bias) were considered.

**Results:**

A total of 309 studies of over 11,000 sources screened were identified to be eligible for inclusion and critically appraised for study quality and risk-of-bias. No relevant guidelines relating to the aims of the present guideline were found. Elected delegates of in total 21 German scientific societies and organizations agreed upon a total of 19 evidence-based statements and recommendations based on a nominal consensus process.

**Conclusions:**

Although most malocclusions can be effectively treated both in the early, late mixed, and permanent dentition, evidence suggests that therapy of a pronounced skeletal or dental class II anomaly can be started early to reduce the risk of dental anterior tooth trauma, whereas in a moderate class II anomaly, therapy can preferably be carried out before or during the pubertal growth peak. Therapy of a skeletal or dental class III anomaly should be started early, as this also reduces the need for later surgery to correct the anomaly. The treatment of a pronounced skeletal or dental transverse anomaly should be started early in the upper jaw in order to utilize the high adaptivity of the maxillary structures in young patients.

**Supplementary Information:**

The online version of this article (10.1007/s00056-022-00409-3) contains supplementary information—the German translation of the article, supplementary tables and references—which is available to authorized users.

## Abstract (de)

**Zweck:**

Der ideale Behandlungszeitpunkt in der Kieferorthopädie wird je nach Art und Ausmaß der vorliegenden Dysgnathie und Malokklusion kontrovers diskutiert, insbesondere im Hinblick auf Effizienz, Patientenbelastung und Behandlungsaufwand einer frühen gegenüber einer regulären oder späten Behandlung. Diese klinische S3-Praxisleitlinie soll klären, zu welchen Zeitpunkten eine kieferorthopädische Anomalie effektiv behandelt werden kann und wie sich die Behandlungseffizienz je nach Behandlungszeitpunkt unterscheidet.

**Methoden:**

Eine systematische Literaturrecherche wurde in verschiedenen Leitliniendatenbanken und den Datenbanken PROSPERO, MEDLINE (PubMed), Cochrane Library, Web of Science, ClinicalTrials.gov und der International Clinical Trials Registry Platform nach dem vordefinierten PICO(Population, Intervention, Comparison and Outcomes with added qualitative search terms)-Suchalgorithmus durchgeführt. Die Bewertung der wissenschaftlichen Evidenz der auf Eignung geprüften Einzelstudien erfolgte mit den Instrumenten SIGN (Scottish Intercollegiate Guidelines Network), AMSTAR II (Assessing the Methodological Quality of Systemic Reviews) und AXIS (Appraisal Tool to Assess the Quality of Cross-sectional Studies). Es wurden nur kontrollierte Studien mit hoher, akzeptabler oder moderater Qualität (und damit einem akzeptablen Bias-Risiko) berücksichtigt.

**Ergebnisse:**

Insgesamt 309 Studien aus über 11.000 gescreenten Quellen wurden als geeignet für die Aufnahme identifiziert und hinsichtlich Studienqualität und Bias-Risiko kritisch bewertet. Es wurden keine relevanten Leitlinien zu den Zielen der vorliegenden Leitlinie gefunden. Gewählte Mandatsträger von insgesamt 21 deutschen Fachgesellschaften und Verbänden verständigten sich auf insgesamt 19 evidenzbasierte Stellungnahmen und Empfehlungen auf Basis eines nominellen Konsensverfahrens.

**Schlussfolgerungen:**

Obwohl die meisten Fehlstellungen sowohl im frühen und späten Wechsel- als auch im bleibenden Gebiss wirksam behandelt werden können, deutet die Evidenz darauf hin, dass die Therapie einer ausgeprägten skelettalen oder dentalen Klasse-II-Anomalie frühzeitig begonnen werden kann, um das Risiko eines dentalen Frontzahntraumas zu reduzieren, während bei einer moderaten Klasse-II-Anomalie die Therapie vorzugsweise vor oder während des pubertären Wachstumsgipfels durchgeführt werden kann. Die Therapie einer skelettalen oder dentalen Klasse-III-Anomalie sollte frühzeitig begonnen werden, da dies auch die Notwendigkeit einer späteren Operation zur Korrektur der Anomalie reduziert. Die Behandlung ausgeprägter transversaler Anomalien sollte insbesondere bei skelettaler Mitbeteiligung oder resultierenden funktionellen Abweichungen frühzeitig im Oberkiefer begonnen werden, um die hohe Adaptivität der Oberkieferstrukturen bei jungen Patienten zu nutzen.

## Introduction

### Background

The background of the present S3 guideline “Ideale Behandlungszeitpunkte kieferorthopädischer Anomalien” (AWMF-Registernummer: 083-038) is that dental malocclusions, skeletal dysgnathia, and various types of orofacial dyskinesia are very frequent worldwide and affect about one in two people (or more). In 2006, about 10.6% of 10-year-old children in Germany were reported to have jaw and tooth position anomalies of a moderate degree, 29.4% pronounced anomalies, and 1.4% severe malpositions. According to a current meta-analysis, class II and class III anomalies occur in the mixed dentition in Europe in 30 and 3% of children, respectively, transverse anomalies in at least 36% (crossbite, midline shifts), and vertical anomalies in about 22% of children, while crowded teeth are present in about 42% of all children with mixed dentition. A German epidemiological study revealed that a frontal crossbite of permanent teeth was registered in 3.4 and 5.1% of children, respectively, although class II anomalies were much more common than class III anomalies. Optimal timing of orthodontic treatment is therefore of high clinical relevance.

Dysgnathia and malocclusion are believed to be associated with various dental and medical conditions. For example, the risk of dental trauma with a class II/1 orthodontic anomaly, an enlarged overjet with a receding mandible, is increased by a factor of 2–3. Restrictions in nasopharyngeal space that lead to sleep apnea can be counteracted with functional orthodontic therapies. Especially in contemporary society, shaped by social networks, children and adolescents are often teased and bullied for their misaligned teeth and their oral appearance. Studies indicate that this could have a negative impact on the development of social skills in dealing with other people, as well as emotional development, self-esteem, and quality of life. It is obvious that an early correction of orthodontic anomalies through orthodontic treatment may have positive effects in these cases and may lead to an improved quality of life. Orthodontics is therefore an integral part of dentofacial diagnostics and therapy at various levels, including the monitoring and correction of disorders in the development of teeth and jaws.

Ideal treatment timing in orthodontics is controversially discussed depending on the type and extent of the dysgnathia and malocclusion present, especially with regard to efficiency, patient burden and treatment efforts of early compared to regular or late treatment. Start of treatment can either take place in the deciduous or early mixed dentition, i.e., before the age of 10 (early treatment), in late mixed dentition or early permanent dentition (regular treatment), or in the permanent dentition after most of the growth is completed (late treatment). Early treatment in the deciduous or early mixed dentition can furthermore be the sole therapy or part of a two-phase treatment strategy, consisting of orthopedic measures during the pubertal growth spurt to correct skeletal dysgnathia (functional orthodontics) or measures to prevent the manifestation or progression of anomalies (e.g., elimination of habits with psychological support, correction of a forced bite), followed by subsequent orthodontic measures to correct dentoalveolar tooth position and dental arch anomalies. In the case of pronounced skeletal anomalies, a combined orthodontic–maxillofacial surgery treatment is carried out after growth is complete.

### Aims of the S3 guideline

The goal of the S3 guideline is the identification and standardization of the ideal treatment timing for orthodontic anomalies considering an individually optimal treatment result, an adequate cost/benefit ratio and minimizing possible risks and therapeutic efforts. Specifically, we aimed to clarify, at which time points an orthodontic anomaly can be effectively treated and how treatment efficiency differs depending on treatment timing.

Two major research questions were addressed for class I (dental crowding), class II, class III, transverse and vertical anomalies according to PICO (Population/Patient, Intervention, Comparison, Outcome):

- In patients with a class I, II, III, transverse or vertical anomaly (P), does early orthodontic treatment or regular/late orthodontic treatment (I) have a medical benefit/harm/harm-preventive benefit compared to no orthodontic treatment (C) in terms of (O) skeletal/dentoalveolar orthodontic treatment outcome, occlusion or chewing function, dentofacial esthetics or soft tissue profile, trauma prophylaxis (dental anterior tooth trauma), oral-health-related quality of life (OHRQoL) and psychological development, breathing (airway space, sleep apnea), swallowing and speaking, prosthetic-conservative restorability of the dentition?
- In patients with a class I, II, III, transverse or vertical anomaly (P), does early orthodontic treatment (I) compared to regular/late orthodontic treatment (C) have a medical benefit/harm/harm-preventive benefit in terms of (O) above-stated outcomes (O) including a reduction in the need for further therapy, patient burden or side effects as well as stability of the treatment result?

### Scope

This guideline is aimed at dentists, specialists in oral and maxillofacial surgery, pediatrics, ear, nose and throat medicine, psychiatry and clinical psychology, i.e., at all disciplines involved in the interdisciplinary treatment of malocclusions and dysgnathia as well as functional disorders of the stomatognathic system. The target group of patients are all patients of all ages who need orthodontic treatment or who want additional treatment in outpatient orthodontic care. No inclusion or exclusion criteria are explicitly defined in order to enable general applicability of the guideline.

## Materials and methods

This S3 Clinical Practice Guideline was developed according to the guidelines of the German Working Group of Scientific Medical Societies AWMF (version 2.0 dated November 19, 2020, http://www.awmf.org/leitlinien/awmf-regelwerk.html), the manual “Systematic Research for Evidence Synthesis and Guidelines” (2nd edition, April 1, 2019, Cochrane Germany Foundation, https://www.cochrane.de/de/literaturrecherche), and the Scottish Intercollegiate Guidelines Network (SIGN) guideline developer’s handbook No. 50 (Edinburgh, https://www.sign.ac.uk/our-guidelines/sign-50-a-guideline-developers-handbook/).

### Systematic literature search

As recommended by the Cochrane Foundation, a systematic search was first carried out in guideline databases on August 1 and 2, 2019, comprising the databases of the AWMF, the Guidelines International Network (G-I-N), TRIP, ÄZQ, SIGN, National Institute for Health and Care Excellence (NICE), KCE Reports of the Belgian Health Care Knowledge Centre and IQWiG according to a predefined search algorithm and strategy adapted to individual databases (Supplementary Table 1). Titles, abstracts, and full texts were screened for eligibility by two of the authors (CK and PP) with disagreements resolved by the third author (CL).

In addition, a systematic literature search was performed in the databases PROSPERO, MEDLINE (PubMed), Cochrane Library (CDRS, CENTRAL, DARE, NHS Economic Evaluation Database, HTA), Web of Science, ClinicalTrials.gov, and the International Clinical Trials Registry Platform according to a predefined PICO search algorithm and strategy adapted to individual databases and research interfaces, especially with regard to keywords, syntax and the documents contained (Supplementary Table 2). Wherever possible, published and validated search filters were used for the guidelines and study designs (method filters). Publications of any date in English or German language were considered. Only guidelines, systematic reviews and meta-analyses, controlled cohort/case–control studies as well as randomized controlled trials (RCTs) were searched. Matching keywords were determined for databases with controlled vocabulary/thesaurus. For the selection of the search terms, the search strategies of existing systematic reviews or review protocols were considered. To identify correlative cross-sectional studies, relevant for assessing associations of orthodontic anomalies with clinical–medical outcomes, a separate systematic literature search for this study type was performed in the MEDLINE database (PubMed) on September 13, 2020 according to a predefined PICO search algorithm and strategy (Supplementary Table 3). Publications of any date in English language were considered. Titles and abstracts were screened for eligibility by two of the authors (CK and PP) with disagreements resolved by the third author (CL). Full texts of remaining articles were screened for eligibility by two investigators varying for individual studies (Supplementary Table 4) with disagreements resolved by one of the authors (CK). Furthermore, manual literature search was performed all international orthodontic journals with an impact factor in 2019 (all issues), comprising the *Journal of Orofacial Orthopedics, American Journal of Orthodontics and Dentofacial Orthopedics, European Journal of Orthodontics, Orthodontics and Craniofacial Research, The Angle Orthodontist, The Korean Journal of Orthodontics, Progress in Orthodontics*, and *Seminars in Orthodontics*. There was no manual evaluation of bibliographies/directories.

### Appraisal of the evidence

Appraisal of scientific evidence (study quality, risk-of-bias) of the individual studies was carried out for randomized controlled clinical studies, cohort studies and case–control studies according to SIGN (Scottish Intercollegiate Guidelines Network; https://www.sign.ac.uk/what-we-do/methodology/checklists/) as well as for meta-analyses and systematic reviews according to AMSTAR II (https://amstar.ca/Amstar-2.php), and for cross-sectional studies according to AXIS (https://bmjopen.bmj.com/content/6/12/e011458.full). Only controlled studies with a high, acceptable, or moderate quality (and thus an acceptable risk of bias) according to SIGN, AXIS, or AMSTAR II were considered within the framework of the guideline.

### Development of statements and recommendations

Elected delegates of in total 21 German scientific societies and organizations (Supplementary Table 5) were invited to a consensus conference on November 3, 2020. The guideline was created with the participation of a patient representative. Patients’ views and preferences were thus identified and incorporated. The statements and recommendations were agreed upon with neutral moderation of an AWMF representative based on a nominal group process. Statements and recommendations were formulated taking into account the specifications of the AWMF and the German Agency for Quality in Medicine (AQuMed*/*ÄZQ). The degree of recommendation (A—strong, B—moderate, 0—weak) is based on the strength of the available evidence, but also take into account the clinical relevance of the outcome parameter, the effect size, and the transferability of study results to the patient target group and the German healthcare system. In order to determine the strength of the consensus, the percentage and absolute number of approvals (approval/disapproval/abstention) were determined with “strong consent” and “consent” corresponding to > 95% and > 75–95% approval.

## Results

The systematic search for available guidelines did not reveal any guidelines with relevant content related to the aims, research questions, or inclusion criteria (PICO) of this guideline.

**Fig. 1:**
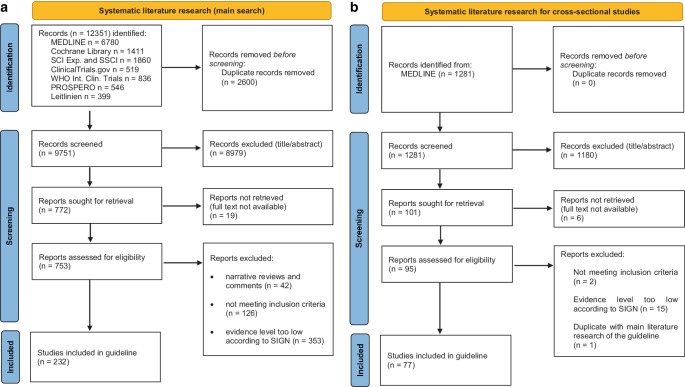
Preferred Reporting Items for Systematic Reviews and Meta-Analyses (PRISMA) flow diagram for the identification and selection of eligible studies. **a** Main systematic literature search, **b** systematic literature search for cross-sectional studies PRISMA(Preferred Reporting Items for Systematic Reviews and Meta-Analyses)-Flussdiagramm zur Identifizierung und Auswahl geeigneter Studien. **a** Systematische Hauptliteraturrecherche, **b** systematische Literaturrecherche für Querschnittsstudien

The main systematic literature search revealed a total of 9751 records after removal of duplicates, of which 8979 were excluded by title and abstract according to the PICO criteria. As full texts of 19 articles could not be retrieved, 753 articles were read and assessed for eligibility. Finally, 232 studies could be included in this guideline (Fig. 1a).

The additional systematic literature search for cross-sectional studies revealed a total of 1281 records, of which 1180 were excluded by title and abstract according to the PICO criteria. As full texts of 6 articles could not be retrieved, 95 articles were read and assessed for eligibility. Finally, 77 correlative studies could be included in this guideline (Fig. 1b).

Due to the complexity and heterogeneity of the available literature, it was unanimously decided at the consensus conference (20/0/0) that the areas vertical anomalies and dental crowding not be addressed in the present version of the guideline and will be taken account in a future update of the guideline after a renewed systematic literature search with close involvement of all professional societies and associations involved in the guideline process.

## Statements and recommendations

### Statement 1: Orthodontic anomalies and mastication

*There is evidence that an orthodontic anomaly can lead to a restriction or suffering with regard to the chewing function.* (consent 18/1/1, level of evidence [LoE] 2+)

### Statement 2: Orthodontic anomalies and oral health-related quality of life

*There are indications that an orthodontic anomaly can lead to a restriction or suffering with regard to oral health-related quality of life (OHRQoL) or psychological development. *(consent 18/1/0, LoE 2+)

### Statement 3: Orthodontic anomalies and disorders of breathing, speaking, and swallowing

*There are indications that there is an association between orthodontic anomalies and disorders of breathing (airway space, sleep apnea), speech, and swallowing. *(consent 19/1/0, LoE 2+/3)

### Statement 4: Orthodontic anomalies and risk of anterior dental trauma

*There is an association between an enlarged dental overjet and an increased risk of anterior dental trauma. There are indications that the absence of proper lip coverage of frontal teeth and a frontal open bite are contributing factors. *(strong consent 21/1/0, LoE 2+)

### Statement 5: Prosthetic-conservative restorability of the dentition

*Prosthetic-conservative restorability of the dentition can be limited in presence of orthodontic anomalies. *(strong consent 21/1/0)

### Statement 6: Orthodontic treatment and oral health-related quality of life

*Depending on the anomaly present, orthodontic treatment leads to an improvement in terms of the methodologically measurable oral health-related quality of life (OHRQoL) compared to no orthodontic treatment.* (strong consent 20/0/1, LoE 1++)

### Statement 7: Class II—early treatment—skeletal, dentoalveolar, and esthetic improvements

*Depending on the intended therapy, early orthodontic treatment of a class II anomaly in the deciduous or early mixed dentition compared to no orthodontic treatment leads to*

- An improvement in the skeletal positional relationship of the maxilla and mandible,
- Dentoalveolar improvements in terms of tooth position, dental arch shape, and masticatory occlusion,
- An improvement of dentofacial esthetics or the soft tissue profile, and
- Improvements in nasopharyngeal and oropharyngeal airway space.

(consent 18/1/0, LoE 1++)

### Statement 8: Class II—early treatment—risk of anterior dental trauma

*Early orthodontic treatment in the deciduous or early mixed dentition can reduce the risk of anterior dental trauma in patients with class II anomaly compared to no orthodontic treatment. *(strong consent 20/1/0, LoE 1++)

### Statement 9: Class II—regular/late treatment—skeletal, dentoalveolar, and esthetic improvements

*Depending on the intended therapy, regular/late orthodontic treatment of a class II anomaly in the late mixed or permanent dentition compared to no orthodontic treatment leads to*

- An improvement in the skeletal positional relationship of the maxilla and mandible,
- Dentoalveolar improvements in terms of tooth position, dental arch shape, and masticatory occlusion, and
- An improvement of dentofacial esthetics or the soft tissue profile.

(consent 18/1/0, LoE 1++)

### Statement 10: Class II—regular/late treatment—disorders of breathing

*In the case of a class II anomaly, regular/late orthodontic treatment in the late mixed or permanent dentition can have positive effects on respiratory disorders (airway space) compared to orthodontic treatment that was not carried out.* (consent 19/0/1, LoE 2+)

### Recommendation 11: Ideal treatment timing of a class II anomaly

*Therapy of a pronounced skeletal or dental class II anomaly can be started early, especially to reduce the risk of a dental anterior tooth trauma or if patient-specific factors are present.*

*In the case of a moderate class II anomaly, therapy in the late mixed dentition can preferably be carried out before or during the pubertal growth peak because the expected skeletal therapy effects are most pronounced at this point in time.*

*In treatments beyond the growth spurt, dentoalveolar therapy effects seem to increasingly dominate treatment, which can also be desirable in individual cases.*

(strong consent 20/0/0, LoE 1++, 0—weak recommendation)

### Statement 12: Class III—early treatment—skeletal, dentoalveolar, and esthetic improvements

*Depending on the intended therapy, early orthodontic treatment of a class III anomaly in the deciduous or early mixed dentition compared to no orthodontic treatment leads to*

- An improvement in the skeletal positional relationship of the maxilla and mandible,
- Dentoalveolar improvements in terms of tooth position, dental arch shape, and masticatory occlusion, and
- An improvement of dentofacial esthetics or the soft tissue profile.

*In addition, there is evidence that maxillary protraction enlarges the upper airway. Overall, (interceptive) orthodontic therapy, possibly supported by skeletal anchorage, seems to be particularly effective in this developmental phase for the correction of skeletal class III. *(consent 18/1/0, LoE 1++)

### Statement 13: Class III—regular/late treatment—skeletal, dentoalveolar, and esthetic improvements

*Depending on the intended therapy, regular/late orthodontic treatment of a class III anomaly in the late mixed or permanent dentition compared to no orthodontic treatment leads to*

- An improvement in the skeletal positional relationship of the maxilla and mandible,
- Dentoalveolar improvements in terms of tooth position, dental arch shape, and masticatory occlusion, and
- An improvement of dentofacial esthetics or the soft tissue profile.

(consent 19/1/0, LoE 1++)

### Statement 14: Class III—regular/late treatment—disorders of breathing

*In the case of a class III anomaly, regular/late orthodontic treatment in the late mixed or permanent dentition can have positive effects on respiratory disorders (airway space) compared to orthodontic treatment that was not carried out. *(consent 18/0/1, LoE 2+)

### Statement 15: Class III—late treatment—surgical bite correction

*The combined orthodontic–surgical correction of a class III malocclusion improves occlusion and facial esthetics. The combined treatment can also improve psychosocial well-being. In addition, there is evidence that mastication can be improved. *(consent 19/0/1, LoE 2++)

### Recommendation 16: Ideal treatment timing of a class III anomaly

*Therapy of a skeletal or dental class III anomaly should be started early, for example in the early mixed dentition phase. There is also evidence that early treatment of a class III anomaly reduces the need for surgery to correct the anomaly. *(strong consent 19/0/0, LoE 1+, B—moderate recommendation)

### Statement 17: Transverse anomalies—early treatment—skeletal, dentoalveolar, esthetic, and breathing improvements

*Depending on the intended therapy, early orthodontic treatment of a transverse anomaly in the deciduous or early mixed dentition compared to no orthodontic treatment leads to*

- An improvement in the skeletal positional relationship of the maxilla and mandible, and
- Dentoalveolar improvements in terms of tooth position, dental arch shape and masticatory occlusion.

*In addition, there is evidence that maxillary expansion enlarges the upper airway.* (consent 19/1/0, LoE 1++)

### Statement 18: Transverse anomalies—regular/late treatment—skeletal, dentoalveolar, esthetic, and breathing improvements

*Depending on the intended therapy, regular/late orthodontic treatment of a transverse anomaly in the late mixed or permanent dentition compared to no orthodontic treatment leads to*

- An improvement in the skeletal positional relationship of the maxilla and mandible, and
- Dentoalveolar improvements in terms of tooth position, dental arch shape, and masticatory occlusion.

*In addition, there is evidence that maxillary expansion enlarges the upper airway.* (consent 19/1/0, LoE 1++)

### Recommendation 19: Ideal treatment timing of a transverse anomaly

*The treatment of a pronounced skeletal or dental transverse anomaly should be started early in the upper jaw in order to utilize the high adaptivity of the maxillary structures in young patients, to counteract muscular malfunctions, and to enable coordinated transverse and sagittal further development of the jaws. *(strong consent 20/0/0, LoE 2++, B—moderate recommendation)

## Conclusions

The present S3 guideline could point out that orthodontic treatment has positive effects on various medical levels, comprising skeletal and dental corrections along with improvement of respiration, positive effects on psychoemotional development and quality of life and preventive effects, e.g., with respect to dental trauma.

With respect to ideal treatment timing, class II anomalies are a heterogeneous group within the field of orthodontics and can be treated differently at different treatment times. Early intervention seems to make sense, especially in the case of very pronounced malpositions and dental overjet because of the subsequent risk of trauma to the upper incisors. Otherwise, class II anomalies have the potential to be effectively treated in the late mixed dentition phase and also in the early permanent dentition. In case of an intervention beyond the pubertal growth peak, there are still promising possibilities for dentoalveolar correction with fixed class II therapies, with skeletal therapy effects becoming increasingly smaller. In specific cases, class II camouflage can also be considered. When growth is complete, there is also the possibility of a surgical correction of the bite position, especially in case of extraoral deviations or due to the complexity of the treatment case (e.g., additional skeletal deviations in other spatial planes)—a purely dentoalveolar correction would in these cases exceed the biological scope of required tooth movements.

Class III anomalies are also a heterogeneous group and can be treated differently at different treatment times. The treatment options in patients with mixed dentition range from simple measures of dentoalveolar correction, for example correction of a frontal crossbite, and interceptive measures for the coordinated further development of the jaws to skeletal orthodontic measures to influence the growth of the upper and lower jaw. There are indications that both dentoalveolar and skeletal therapeutic measures should be started early, e.g., in the early mixed dentition, in order to be able to fully utilize the potential for a positive growth influence, especially in the upper jaw. Activation protocols such as Alt-RAMEC (alternate rapid maxillary expansions and constrictions) can, if necessary using skeletal anchoring techniques, effectively expand the therapeutic spectrum in the early and late mixed dentition. However, orthodontics can also make an important contribution beyond the optimal timepoint for treatment: in mild cases, dentoalveolar compensation can be considered, and in patients with pronounced skeletal malpositions of the jaws, a combined orthodontic–maxillofacial correction can be successfully performed after growth has ended depending on the quality of orthodontic pre- and posttreatment.

The treatment of a pronounced skeletal or dental transverse anomaly can be started early in the upper jaw in order to utilize the high adaptivity of the maxillary structures in the young patient, to counteract muscular malfunctions, and to enable a coordinated further transverse and sagittal jaw development.

## Supplementary Information

German translation

Supplementary Table 1: Search string used for guideline databases (here adapted to the database National Institute for Health and Care Excellence NICE)

Supplementary Table 2: Search string and history used for the main systematic literature search (here adapted to the database MEDLINE/PubMed)

Supplementary Table 3: Search string and history used for the additional systematic literature search for cross-sectional studies (MEDLINE/PubMed)

Supplementary Table 4: Scientific investigators involved in the eligibility screening and quality assessment of full-text articles

Supplementary Table 5: Elected delegates of the 21 German scientific societies, who participated in the development and consensus of statements and recommendations

References

