# Supplementary material for: Ideal treatment timing of orthodontic anomalies—a German clinical S3 practice guideline
Source: J Orofac Orthop. 2022 Jun 17;83(4):225–32. doi: 10.1007/s00056-022-00409-3 (PMC9226101; doi:10.1007/s00056-022-00409-3)
Supplement: Supplementary file 2 — Supplementary Table 1: Search string used for guideline databases (here adapted to the database National Institute for Health and Care Excellence NICE) [file 56_2022_409_MOESM2_ESM.pdf]

Supplementary Table 1: Search string used for guideline databases (here adapted to the database National Institute for Health and Care Excellence NICE).

Ergänzungstabelle 1: Suchalgorithmus für Leitliniendatenbanken (hier angepasst an die Datenbank National Institute for Health and Care Excellence NICE).

orthodont* OR orthognath* OR malocclu* OR retrognath* OR micrognath* OR prognath* OR "angle class" OR "class i" OR "class ii" OR "class iii" OR occlusion* OR bite OR retrognath* OR micrognath* OR overjet OR "over jet" OR prognath* OR progeny OR cleft OR Engstand OR "facial asymmetry" OR crossbite OR "cross bite" OR "non occlusion" OR nonocclusion OR crowding OR "open bite" OR openbite OR "deep bite" OR deepbite OR "over bite" OR overbite OR "under bite" OR underbite OR "reverse bite" OR reversebite OR "midline deviation" OR "midline shift" OR "mandibular deviation" OR dentofacial OR "dento facial" OR "tooth movement" OR "craniofacial growth" OR "growth modification" OR multibracket OR "fixed appliance" OR "fixed appliances" OR "functional appliance" OR "functional appliances"
