# Supplementary material for: Ideal treatment timing of orthodontic anomalies—a German clinical S3 practice guideline
Source: J Orofac Orthop. 2022 Jun 17;83(4):225–32. doi: 10.1007/s00056-022-00409-3 (PMC9226101; doi:10.1007/s00056-022-00409-3)
Supplement: Supplementary file 3 — Supplementary Table 2: Search string and history used for the main systematic literature search (here adapted to the database MEDLINE/PubMed) [file 56_2022_409_MOESM3_ESM.pdf]

Supplementary Table 2: Search string and history used for main systematic literature research (here adapted to the database MEDLINE/PubMed).

Ergänzungstabelle 2: Suchalgorithmus und -historie für die systematische Hauptliteraturrecherche (hier angepasst an die Datenbank MEDLINE/PubMed).

Step	Search string	Hits	Comment
1	Malocclusion, Angle Class I/ or Open Bite/ or (Malocclusion/ and anterior$.ti,ab,kf.) or (("class i" or "class 1") and (angle or angles or malocclusion$ or bite$)).ti,ab,kf. or (openbite$ or "open bite$" or apertognathi$ or nonocclusion$ or non-occlusion$).ti,ab,kf. or (crossbite$ or "cross bite$").ti,ab,kf. or (overbite$ or "over bite$").ti,ab,kf. or (underbite$ or "under bite$").ti,ab,kf. or (deepbite$ or "deep bite$").ti,ab,kf. or Engstand.ti,ab,kf. or crowding.ti,ab,kf. or Facial Asymmetry/ or "facial asymmetr$".ti,ab,kf. or ("midline deviation$" or "midline shift$" or "mandibular deviation$").ti,ab,kf. or "short face syndrome$".ti,ab,kf.	25081	Angle class I malocclusion
2	exp Malocclusion, Angle Class II/ or Retrognathism/ or Micrognathism/ or (("class ii" or "class 2") and (angle or angles or malocclusion$ or bite$)).ti,ab,kf. or (posterior adj3 occlusion$).ti,ab,kf. or (distoclusion$ or disto-occlusion$ or distocclusion$).ti,ab,kf. or (distal adj (occlusion$ or bite$)).ti,ab,kf. or retrognath$.ti,ab,kf. or (prominent adj3 upper adj3 teeth).ti,ab,kf. or (overjet$ or "over jet$").ti,ab,kf. or (mandib$ adj3 (micrognath$ or retropos$ or retrus$)).ti,ab,kf. or (maxill$ adj3 (prognath$ or anteposition$)).ti,ab,kf. or ((teeth or incisor$) adj3 (retro-clin$ or retroclin$)).ti,ab,kf.	13837	Angle class II malocclusion
3	Malocclusion, Angle Class III/ or Prognathism/ or (("class III" or "class 3") and (angle or angles or malocclusion$ or bite$)).ti,ab,kf. or "reverse bite$".ti,ab,kf. or prognath$.ti,ab,kf. or (prominent adj3 lower adj3 teeth).ti,ab,kf. or ("mesial occlusion$" or "mesial bite$").ti,ab,kf. or (progeny and (chin or face or maxillofac$ or orthodontic$)).ti,ab,kf. or ("negative overjet" or "negative over-jet").ti,ab,kf. or (maxill$ adj3 (micrognath$ or retropos$ or retrus$)).ti,ab,kf. or Cleft Palate/ or Cleft Lip/ or (harelip$ or "hare lip$").ti,ab,kf. or (cleft adj3 (lip$ or palat$)).ti,ab,kf.	36427	Angle class III malocclusion

4	Malocclusion/ or malocclusion$.ti,ab,kf. or Dental Occlusion, Traumatic/ or "traumatic dental occlusion".ti,ab,kf. or ("angle class$" or "angles class$").ti,ab,kf.	31559	Malocclusion unspecified
5	or/1-4	83640	Malocclusion total
6	exp Orthodontics/ or ((orthodontic$ or dental) and (brace$ or band$ or wire$)).ti,ab,kf. or (orthodontic$ and (extract$ or remov$)).ti,ab,kf. or (orthodontic$ and (headgear$ or "head gear$")).ti,ab,kf. or ((appliance$ or device$) adj5 (function$ or remova$ or fix$)).ti,ab,kf. or ((appliance$ or device$) adj5 (intraoral or "intra oral" or extraoral or "extra oral")).ti,ab,kf. or (activator adj appliance$).ti,ab,kf. or (Frankel or "twin$ block$" or FR-II).ti,ab,kf. or ((growth adj3 modif$) and (jaw$ or maxilla$ or mandible$ or mandibular)).ti,ab,kf. or ((one-phase or two-phase) and (treatment or therapy) and (orthodontic$ or malocclusion$)).ti,ab,kf. or ((extraoral or "extra oral") and traction).ti,ab,kf. or "chin cap$".ti,ab,kf. or (("face mask$" or facemask$ or "reverse head-gear" or "reverse headgear") and orthodontic$).ti,ab,kf. or ((orthopedic$ or orthopaedic$) and (dental or orthodontic$ or facial)).ti,ab,kf. or (orthodontic$ adj (alignment$ or correction$ or therapy or therapies or treatment$ or management or interceptive or interception$ or rehabilitation)).ti,ab,kf. or ("tooth movement" or OTM).ti,ab,kf. or (intrusion$ or extrusion$ or "mesial movement$" or "distal movement$" or protraction$).ti,ab,kf. or ("maxillary expansion$" or "arch expansion$" or (RME and maxill$) or SARME).ti,ab,kf. or ((camouflage or advancement or repositioning) and orthodont$).ti,ab,kf.	108585	Non-surgical orthodontic treatment
7	exp orthognathic surgical procedures/ or osteotomy, le fort/ or osteotomy, sagittal split ramus/ or Osteotomy/ or Surgery, Oral/ or Oral Surgical Procedures/ or Jaw Fixation Techniques/ or Mandibular Advancement/ or exp Orthognathic Surgical Procedures/ or ((Jaw Abnormalities/ or exp Malocclusion/ or Micrognathism/ or Retrognathia/ or exp Jaw/) and su.fs.) or orthognath$.ti,ab,kf. or (orthodont$ and	103767	Orthognathic surgery

	(surgery or surgeries or surgical)).ti,ab,kf. or ((mandib$ or jaw$ or maxill$ or bimaxillary or Prognath$ or Retrognath$ or occlusion or malocclusion or angle or "class I" or "class 1" or "class II" or "class 2" or "class III" or "class 3" or nonocclusion or overbite$ or "cross bite$" or crossbite$ or "under bite$" or underbite$ or "over bite$" or openbite$ or "open bite$" or "deep bite$" or deepbite$ or "over jet$" or overjet$ or dentofacial or "dento facial" or ramal or ramus) adj3 (surg$ or procedure$ or operation$ or operative or correct$ or osteotom$ or advancement or reduction$ or reposition$ or setback or advancement or retropos$ or retrus$ or fixation$ or distract$ or resect$)).ti,ab,kf. or (mandibulotom$ or mandibulectom$ or hemimandibulectom$ or maxillotom$ or maxillectom$).ti,ab,kf. or ("Le Fort" or lefort).ti,ab,kf. or ((jaw abnormalities/ or micrognathism/ or retrognathia/ or malocclusion/) and su.fs.)		
8	6 or 7	197917	Orthodontic treatment
9	5 and 8	32712	Malocclusion total and orthodontic treatment
10	exp clinical pathway/ or exp clinical protocol/ or exp consensus/ or exp consensus development conference/ or exp consensus development conferences as topic/ or critical pathways/ or exp guideline/ or guidelines as topic/ or exp practice guideline/ or practice guidelines as topic/ or health planning guidelines/ or exp treatment guidelines/ or (guideline or practice guideline or consensus development conference or consensus development conference, NIH).pt. or (position statement* or policy statement* or practice parameter* or best practice*).ti,ab,kf,kw. or (standards or guideline or guidelines).ti,kf,kw. or ((practice or treatment* or clinical) adj guideline*).ab. or (CPG or CPGs).ti. or consensus*.ti,kf,kw.	470124	
11	consensus*.ab. /freq=2	23244	
12	((critical or clinical or practice) adj2 (path or paths or pathway or pathways or protocol*)).ti,ab,kf,kw. or	124175	

	recommendat*.ti,kf,kw. or (care adj2 (standard or path or paths or pathway or pathways or map or maps or plan or plans)).ti,ab,kf,kw. or (algorithm* adj2 (screening or examination or test or tested or testing or assessment* or diagnosis or diagnoses or diagnosed or diagnosing)).ti,ab,kf,kw. or (algorithm* adj2 (pharmacotherap* or chemotherap* or chemotreatment* or therap* or treatment* or intervention*)).ti,ab,kf,kw.		
13	or/10-12	577944	CADTH Search Filter for Guidelines - OVID Medline, Embase, PsycINFO
14	9 and 13	429	Malocclusion total AND orthodontic treatment AND Guidelines
15	meta-analysis.pt. or (meta-analysis/ or systematic review/ or meta-analysis as topic/ or "meta analysis (topic)"/ or "systematic review (topic)"/ or exp technology assessment, biomedical/) or ((systematic* adj3 (review* or overview*)) or (methodologic* adj3 (review* or overview*))).ti,ab,kf,kw. or ((quantitative adj3 (review* or overview* or synthes*)) or (research adj3 (integrati* or overview*))).ti,ab,kf,kw. or ((integrative adj3 (review* or overview*)) or (collaborative adj3 (review* or overview*)) or (pool* adj3 analy*)).ti,ab,kf,kw. or (data synthes* or data extraction* or data abstraction*).ti,ab,kf,kw. or (handsearch* or hand search*).ti,ab,kf,kw. or (mantel haenszel or peto or der simonian or dersimonian or fixed effect* or latin square*).ti,ab,kf,kw. or (met analy* or metanaly* or technology assessment* or HTA or HTAs or technology overview* or technology appraisal*).ti,ab,kf,kw. or (meta regression* or metaregression*).ti,ab,kf,kw. or (meta-analy* or metaanaly* or systematic review* or biomedical technology	422572	CADTH Search Filter for Systematic Reviews/Meta-Analysis/Health Technology Assessment – OVID Medline, Embase

	assessment* or bio-medical technology assessment*).mp,hw. or (medline or cochrane or pubmed or medlars or embase or cinahl).ti,ab,hw. or (cochrane or (health adj2 technology assessment) or evidence report).jw. or (comparative adj3 (efficacy or effectiveness)).ti,ab,kf,kw. or (outcomes research or relative effectiveness).ti,ab,kf,kw. or ((indirect or indirect treatment or mixed-treatment) adj comparison*).ti,ab,kf,kw.		
16	9 and 15	498	Malocclusion total AND orthodontic treatment AND SR & meta-analyses & HTA
17	14 or 16	914	Malocclusion total AND orthodontic treatment AND Guidelines & SR & meta-analyses & HTA
18	limit 17 to (english or german)	859	Language filter
19	randomized controlled trial.pt.	486827	
20	controlled clinical trial.pt.	93200	
21	randomized.ab.	451813	
22	placebo.ab.	200030	
23	clinical trials as topic.sh.	187884	
24	randomly.ab.	316380	
25	trial.ti.	203268	
26	19 or 20 or 21 or 22 or 23 or 24 or 25	1232705	
27	exp animals/ not humans.sh.	4606297	
28	26 not 27	1133858	Cochrane Highly Sensitive Search Strategy 2008 sensitive and precise MEDLINE Ovid

29	Clinical Trial, Phase III/ or ("phase 3" or "phase3" or "phase III" or P3 or "PIII").ti,ab,kw.	68710	Filter P3 for Phase III Clinical Trials. From: Cooper C, Varley-Campbell J, Carter P. Established search filters may miss studies when identifying randomized controlled trials. Journal of Clinical Epidemiology. 2019;112:12–9. doi: 10.1016/j.jclinepi.2019.04.002. PubMed PMID: 30986533.
30	9 and 28	1449	Malocclusion total AND orthodontic treatment AND study filter Cochrane
31	9 and 29	21	Malocclusion total AND orthodontic treatment AND P3-study-filter
32	30 or 31	1466	Malocclusion total AND orthodontic

			treatment AND primary studies
33	limit 32 to (english or german)	1386	Language filter
34	(effectiveness or efficacy or effect or effects or impact or benefit or benefits or advantage or advantages or value or usefulness or avail or avails or improve$ or superior or superiority or inferior or inferiority or "treatment result" or "treatment results" or outcome or outcomes or need or preventive or prevention or duration or "treatment time" or "health gain" or disadvantage or disadvantages or harm or harms or "side effect" or "side effects" or "adverse effect" or "adverse effects").ti,ab,kf. not (exp animals/ not humans.sh.)	8669135	Comparative view of outcome
35	9 and 34	12636	Malocclusion total AND orthodontic treatment AND comparative view of outcome
36	(early or late or timing or time or deciduous or "primary dentition" or "mixed dentition" or "permanent dentition" or growing or infants or newborn or child or children or teenager or teenagers or adolescent or adolescents or adult or adults).ti,ab,kf.	6501161	Comparison time point of treatment
37	35 and 36	6150	Malocclusion total AND orthodontic treatment AND comparative view of outcome AND comparison time point of treatment
38	limit 37 to (english or german)	5576	Language filter
39	18	859	Aggregated evidence

40	33 not 18	1222	Controlled studies
41	38 not (18 or 33)	4699	Remaining studies on comparison time point of treatment
42	or/39-41	6780	Total hits
