# Supplementary material for: Ideal treatment timing of orthodontic anomalies—a German clinical S3 practice guideline
Source: J Orofac Orthop. 2022 Jun 17;83(4):225–32. doi: 10.1007/s00056-022-00409-3 (PMC9226101; doi:10.1007/s00056-022-00409-3)
Supplement: Supplementary file 4 — Supplementary Table 3: Search string and history used for the additional systematic literature search for cross-sectional studies (MEDLINE/PubMed) [file 56_2022_409_MOESM4_ESM.pdf]

Supplementary Table 3: Search string and history used for the additional systematic literature research for cross-sectional studies (MEDLINE/PubMed).

Ergänzungstabelle 3: Suchalgorithmus und -historie für die zusätzliche systematische Literaturrecherche nach Assoziationsstudien (MEDLINE/PubMed).

Step	Search string	Hits	Comments
1	(orthodont* OR orthognath* OR malocclu* OR dysgnath* OR "angle class" OR "dental occlusion"~3 OR "distal occlusion" OR "posterior occlusion" OR "mesial occlusion" OR "prominent lower front teeth" OR "prominent upper front teeth" OR retrognath* OR micrognath* OR overjet OR "over jet" OR prognath* OR "facial asymmetry" OR crossbite OR "cross bite" OR "non occlusion" OR nonocclusion OR "open bite" OR openbite OR "deep bite" OR deepbite OR "mesial bite" OR "over bite" OR overbite OR "under bite" OR underbite OR "reverse bite" OR (crowding AND (tooth OR teeth OR dental)) OR "midline deviation" OR "midline shift" OR "mandibular deviation" OR "tooth movement" OR "craniofacial growth" OR "growth modification" OR multibracket OR "fixed appliance" OR "fixed appliances" OR "functional appliance" OR "functional appliances" OR ((extraoral OR "extra oral") AND traction) OR chincap OR "chin cap" OR chincaps OR "chin caps")	59.793	Malocclusions and dysgnathias, orthodontic treatment
2	(correlation* OR association* OR relation* OR interrelation* OR connection* OR interconnection* OR link)	4.885.006	associations
3	("quality of life" OR QoL OR OHRQoL OR psycholog*)	1.967.862	Quality of life
4	(mastication OR masticator* OR gastric)	431.891	Mastication and digestion
5	(airway OR apnea OR sleep OR respiration)	630.699	Respiration, sleep, airway
6	swallow*	32.545	Swallowing
7	(speech OR sigmatism OR phoneti* OR phonat*)	136.434	Speaking

8	(trauma* AND (tooth OR teeth OR dental OR incisor OR incisal))	16.483	Dental Trauma
9	1 AND 2	12.002	Associations with Malocclusions and dysgnathias, orthodontic treatment (AMDOT)
10	9 AND 3	921	AMDOT AND quality of life
11	9 AND 4	861	AMDOT AND Mastication and digestion
12	9 AND 5	646	AMDOT AND Respiration, sleep, airway
13	9 AND 6	138	AMDOT AND Swallowing
14	9 AND 7	293	AMDOT AND Speaking
15	9 AND 8	781	AMDOT AND dental trauma
16	(review* OR overview* OR synthes* OR meta* OR RCT OR randomized controlled trial OR cohort OR case-control OR "case control")		Reviews/RCTs/controlled studies
17	9 AND 3 AND 16	266	Reviews/RCTs/controlled studies AND AMDOT AND quality of life
18	9 AND 4 AND 16	290	Reviews/RCTs/controlled studies AND Mastication and digestion
19	9 AND 5 AND 16	305	Reviews/RCTs/controlled studies AND Respiration, sleep, airway

20	9 AND 6 AND 16	46	Reviews/RCTs/controlled studies AND Swallowing
21	9 AND 7 AND 16	101	Reviews/RCTs/controlled studies AND Speaking
22	9 AND 8 AND 16	273	Reviews/RCTs/controlled studies AND dental trauma
