# Supplementary material for: Ideal treatment timing of orthodontic anomalies—a German clinical S3 practice guideline
Source: J Orofac Orthop. 2022 Jun 17;83(4):225–32. doi: 10.1007/s00056-022-00409-3 (PMC9226101; doi:10.1007/s00056-022-00409-3)
Supplement: Supplementary file 5 — Supplementary Table 4: Scientific investigators involved in the eligibility screening and quality assessment of full-text articles [file 56_2022_409_MOESM5_ESM.pdf]

Supplementary Table 4: Scientific investigators involved in the eligibility screening and quality assessment of full-text articles.

Ergänzungstabelle 4: Prüf(zahn)ärzte, die an der Überprüfung der Eignung und der Qualitätsbewertung von Volltextartikeln beteiligt waren.

Scientific society	Abbreviation	Investigator
**Working group I: class II anomalies**		
German Orthodontic Society	DGKFO	Priv.-Doz. Dr. Dr. Christian Kirschneck (Sprecher, Regensburg)
German Orthodontic Society	DGKFO	Dr. Julia von Bremen (Gießen)
German Orthodontic Society	DGKFO	Prof. Dr. Angelika Stellzig-Eisenhauer (Würzburg)
German Orthodontic Society	DGKFO	Priv.-Doz. Dr. Felix Kunz (Würzburg)
German Orthodontic Society	DGKFO	Dr. Lena Goetz (Würzburg)
German Orthodontic Society	DGKFO	Dr. Stefan Keß (Würzburg)
German Orthodontic Society	DGKFO	ZÄ Lisa Marie Widmaier (Würzburg)
German Orthodontic Society	DGKFO	Dr. Carolin Ziegler (Würzburg)
German Orthodontic Society	DGKFO	ZÄ Anja Kunz (Würzburg)
German Orthodontic Society	DGKFO	Dr. Maximilian Bock (Würzburg)
German Orthodontic Society	DGKFO	Dr. Jana Grote (Würzburg)
German Orthodontic Society	DGKFO	Dr. Elisabeth Hübers (Würzburg)
German Orthodontic Society	DGKFO	Priv.-Doz. Dr. Agnes Schröder (Regensburg)
German Orthodontic Society	DGKFO	Dr. Eva Paddenberg (Regensburg)
German Orthodontic Society	DGKFO	Dr. Jonas Breunig (Regensburg)

German Orthodontic Society	DGKFO	Dr. Niklas Ullrich (Regensburg)
German Society for Operative Dentistry	DGZ	Prof. Dr. Anahita Jablonski-Momeni (Marburg)
German Society for Preventive Dentistry	DGPZM	Dr. Lutz Laurisch (Korschenbroich)
**Working group I: class III anomalies**		
German Orthodontic Society	DGKFO	Prof. Dr. Christopher J. Lux (Sprecher, Heidelberg)
German Orthodontic Society	DGKFO	Dr. Ralf Erber (Heidelberg)
German Orthodontic Society	DGKFO	Dr. Sinan Şen (Heidelberg)
German Orthodontic Society	DGKFO	Dr. Christoph Roser (Heidelberg)
German Orthodontic Society	DGKFO	Priv.-Doz. Dr. Kathrin Becker (Düsseldorf)
German Society for Medical Psychology	DGMP	Dr. Jutta Margraf-Stiksrud (Marburg)
German Society for Oral and Maxillofacial Surgery	DGMKG	Prof. Dr. Dr. Christian Freudlsperger (Heidelberg)
**Working group III: transversal and vertical anomalies, dental crowding**		
German Orthodontic Society	DGKFO	Prof. Dr. Dr. Ralf J. Radlanski (Sprecher, Berlin)
German Orthodontic Society	DGKFO	Prof. Dr. Philipp Meyer-Marcotty (Göttingen)
German Orthodontic Society	DGKFO	Dr. Anja Quast (Göttingen)
German Orthodontic Society	DGKFO	Dr. Daniela Klenke (Göttingen)

German Orthodontic Society	DGKFO	Dr. Petra Santander (Göttingen)
German Orthodontic Society	DGKFO	Dr. Sarah Batschkus (Göttingen)
German Orthodontic Society	DGKFO	ZA Bernhard Wiechens (Göttingen)
German Orthodontic Society	DGKFO	Dr. Florian Behrend (Göttingen)
German Society for Otorhinolaryngology, Head- and neck surgery	DGHNO KHC	Prof. Dr. med. Mark Praetorius (Mainz)
Working group for oral and maxillofacial surgery	AgKi	Patricia Parvini (Kassel)
Federal Association of Pediatric Dentists	BUKiZ	Dr. Monika Prinz-Kattinger (Bad Aibling)
German Society for Paediatric Dentistry	DGKiZ	Dr. Sabine Dobersch-Paulus (Würzburg)
