# Supplementary material for: Ideal treatment timing of orthodontic anomalies—a German clinical S3 practice guideline
Source: J Orofac Orthop. 2022 Jun 17;83(4):225–32. doi: 10.1007/s00056-022-00409-3 (PMC9226101; doi:10.1007/s00056-022-00409-3)
Supplement: Supplementary file 6 — Supplementary Table 5: Elected delegates of the 21 German scientific societies, who participated in the development and consensus of statements and recommendations [file 56_2022_409_MOESM6_ESM.pdf]

Supplementary Table 5: Elected delegates of the 21 German scientific societies, who participated in the development and consensus of statements and recommendations.

Ergänzungstabelle 5: Gewählte Mandatsträger der 21 deutschen wissenschaftlichen Fachgesellschaften, die an der Entwicklung und Abstimmung von Stellungnahmen und Empfehlungen mitgewirkt haben.

**Scientific society**	**Abbreviation**	**Elected Delegates**
**Registering societies**		
German Orthodontic Society	DGKFO	Prof. Dr. Dr. Peter Proff (delegate)<br>Prof. Dr. Christopher J. Lux (coordinator)<br>Priv.-Doz. Dr. Dr. Christian Kirschneck (coordinator)
German Dental Society	DGZMK	Dr. Anke Weber (no vote)
**Scientific Societies of the AWMF**		
German Society for Otorhinolaryngology, Head- and neck surgery	DGHNO KHC	Prof. Dr. med. Mark Praetorius
German Society for Pediatric Dentistry	DGKiZ	Dr. Sabine Dobersch-Paulus
German Society for Pediatric and Adolescent Medicine	DGKJ	Dr. Burkhard Lawrenz
German Society for Child and Adolescent Psychiatry, Psychosomatic Medicine and Psychotherapy	DGKJP	Prof. Dr. Romuald Brunner
German Society for Oral and Maxillofacial Surgery	DGMKG	Prof. Dr. Dr. Christian Freudlsperger

German Society for Medical Psychology	DGMP	Dr. Jutta Margraf-Stiksrud
German Society for Periodontology	DG PARO	Priv.-Doz. Dr. Christian Graetz
German Society for Prosthetic Dentistry and biomaterials	DGPro	Prof. Dr. Marc Schmitter
German Society for Operative Dentistry	DGZ	Prof. Dr. Anahita Jablonski-Momeni
**Further Scientific Societies**		
Working group for basic research in the DGZMK	AfG	Prof. Dr. Dr. Ralf J. Radlanski
Working group for oral and maxillofacial surgery	AGOKi	Patricia Parvini
Federal Working Group of Patient Centers and Initiatives	BAGP	Gregor Bornes
Federal Association of Pediatric Dentists	BUKiZ	Dr. Monika Prinz-Kattinger
Federal Association of Dentists in the Public Health Service (BZÖG)	BZÖG	Dr. Pantelis Petrakakis
German Society for Aesthetic Dentistry	DGÄZ	Prof. Dr. Angelika Stellzig-Eisenhauer
German Society for Preventive Dentistry	DGPZM	Dr. Lutz Laurisch
Interdisciplinary working group on oral pathology and oral medicine	AKOPOM	Prof. Dr. Dr. Urs Müller-Richter, FEBOMFS

National Association of Statutory Health Insurance Dentists	KZBV	Dr. Birgit Lange-Lenz
Association of Medical Professionals	VMF	Sylvia Gabel
