# Supplementary material for: Ideal treatment timing of orthodontic anomalies—a German clinical S3 practice guideline
Source: J Orofac Orthop. 2022 Jun 17;83(4):225–32. doi: 10.1007/s00056-022-00409-3 (PMC9226101; doi:10.1007/s00056-022-00409-3)
Supplement: Supplementary file 7 — References [file 56_2022_409_MOESM7_ESM.pdf]

References

1. Abdalla Y, Brown L, Sonnesen L (2019) Effects of rapid maxillary expansion on upper airway volume: A three-dimensional cone-beam computed tomography study. Angle Orthodontist 019. doi: 10.2319/101218-738.1
2. Abrahamsson C (2013) Masticatory function and temporomandibular disorders in patients with dentofacial deformities. Swedish Dental Journal Supplement(231): 9–85
3. Abu Alhaija ES, Richardson A (1999) Long-term effect of the chincap on hard and soft tissues. Eur. J. Orthodont. 21(3): 291–298
4. Abu Alhaija, Elham S. J., Al-Khateeb SN (2011) Skeletal, dental and soft tissue changes in Class III patients treated with fixed appliances and lower premolar extractions. Aust. Orthod. J. 27(1): 40–45
5. Agirnasligil MO, Amuk NG, Kilic E et al. (2019) The changes of self-esteem, sensitivity to criticism, and social appearance anxiety in orthognathic surgery patients: A controlled study. Am. J. Orthod. Dentofac. Orthop. 155(4): 482-+. doi: 10.1016/j.ajodo.2018.05.019
6. Agostino P, Ugolini A, Signori A et al. (2014) Orthodontic treatment for posterior crossbites. [Review]. Cochrane Database of Systematic Reviews 014(8): CD000979. doi: 10.1002/14651858.CD000979.pub2
7. Agou S, Locker D, Muirhead V et al. (2011) Does psychological well-being influence oral-health-related quality of life reports in children receiving orthodontic treatment? American Journal of Orthodontics & Dentofacial Orthopedics 139(3): 369–377. doi: 10.1016/j.ajodo.2009.05.034
8. Alali OH (2014) A prospective controlled evaluation of Class II division 1 malocclusions treated with fixed lingual mandibular growth modificator. Angle Orthodontist 84(3): 527–533. doi: 10.2319/070913-500.1
9. Alarcon JA, Bastir M, Rosas A et al. (2011) Chincup treatment modifies the mandibular shape in children with prognathism. American Journal of Orthodontics & Dentofacial Orthopedics 140(1): 38–43. doi: 10.1016/j.ajodo.2009.10.046
10. Al-Jewair TS, Preston CB, Moll EM et al. (2012) A comparison of the MARA and the AdvanSync functional appliances in the treatment of Class II malocclusion. Angle Orthodontist 82(5): 907–914. doi: 10.2319/090411-569.1
11. Al-Khalifa HN, Hashem MI, Alanazi KJ et al. (2017) Orthopedic Effect of Chin Cup during Mixed Dentition Stage. Journal of Contemporary Dental Practice [Electronic Resource] 18(5): 410–414
12. Allen RA, Connolly IH, Richardson A (1993) Early treatment of Class III incisor relationship using the chincap appliance. European Journal of Orthodontics 15(5): 371–376
13. de Almeida, Henriques JF, Almeida RR de et al. (2005) Short-term treatment effects produced by the Herbst appliance in the mixed dentition. Angle Orthodontist 75(4): 540–547. doi: 10.1043/0003-3219(2005)75[540:STEPBT]2.0.CO
14. Almeida-Pedrin RR, Almeida MR, Almeida RR et al. (2007) Treatment effects of headgear biteplane and bionator appliances. American Journal of Orthodontics & Dentofacial Orthopedics 132(2): 191–198

15. Altug-Atac AT, Erdem D (2007) Effects of three-dimensional bimetric maxillary distalizing arches and cervical headgear on dentofacial structures. European Journal of Orthodontics 29(1): 52–59

16. Andiappan M, Gao W, Bernabe E et al. (2015) Malocclusion, orthodontic treatment, and the Oral Health Impact Profile (OHIP-14): Systematic review and meta-analysis. [Review]. Angle Orthodontist 85(3): 493–500. doi: 10.2319/051414-348.1

17. Angelieri F, Franchi L, Cevidanes LH et al. (2014) Long-term treatment effects of the FR-2 appliance: a prospective evalution 7 years post-treatment. European Journal of Orthodontics 36(2): 192–199. doi: 10.1093/ejo/cjt026

18. Anne,Mandall,N, Cousley R, DiBiase A et al. (2012) Is early Class III protraction facemask treatment effective? A multicentre, randomized, controlled trial: 3-year follow-up. Journal of Orthodontics 39(3): 176–185. doi: 10.1179/1465312512Z.00000000028

19. Anthony SN, Zimba K, Subramanian B (2018) Impact of Malocclusions on the Oral Health-Related Quality of Life of Early Adolescents in Ndola, Zambia. International Journal of Dentistry 2018: 7920973. doi: 10.1155/2018/7920973

20. Arraj GP, Rossi-Fedele G, Doğramacı EJ (2019) The association of overjet size and traumatic dental injuries-A systematic review and meta-analysis. Dental Traumatology 35(4-5): 217–232. doi: 10.1111/edt.12481

21. Atik E, Gorucu-Coskuner H, Kocadereli I (2017) Dentoskeletal and airway effects of the X-Bow appliance versus removable functional appliances (Frankel-2 and Trainer) in prepubertal Class II division 1 malocclusion patients. Aust. Orthod. J. 33(1): 3–13

22. Baccetti T, De,Clerck,HJ, Cevidanes LH et al. (2011) Morphometric analysis of treatment effects of bone-anchored maxillary protraction in growing Class III patients. European Journal of Orthodontics 33(2): 121–125. doi: 10.1093/ejo/cjq170

23. Baccetti T, Franchi L, Cameron CG et al. (2001) Treatment timing for rapid maxillary expansion. Angle Orthod. 71(5): 343–350

24. Baccetti T, Franchi L, McNamara,JA,Jr (1999) Thin-plate spline analysis of treatment effects of rapid maxillary expansion and face mask therapy in early Class III malocclusions. European Journal of Orthodontics 21(3): 275–281

25. Baccetti T, Franchi L, McNamara,JA,Jr (2000) Treatment and posttreatment craniofacial changes after rapid maxillary expansion and facemask therapy. American Journal of Orthodontics & Dentofacial Orthopedics 118(4): 404–413

26. Baccetti T, Franchi L, Toth LR et al. (2000) Treatment timing for Twin-block therapy. American Journal of Orthodontics & Dentofacial Orthopedics 118(2): 159–170

27. Baccetti T, McGill JS, Franchi L et al. (1998) Skeletal effects of early treatment of Class III malocclusion with maxillary expansion and face-mask therapy. American Journal of Orthodontics & Dentofacial Orthopedics 113(3): 333–343

28. Baccetti T, Rey D, Oberti G et al. (2009) Long-term outcomes of Class III treatment with mandibular cervical headgear followed by fixed appliances. Angle Orthodontist 79(5): 828–834. doi: 10.2319/111408-580.1

29. Baccetti T, Tollaro I (1998) A retrospective comparison of functional appliance treatment of Class III malocclusions in the deciduous and mixed dentitions. European Journal of Orthodontics 20(3): 309–317

30. Baik HS, Jee SH, Lee KJ et al. (2004) Treatment effects of Frankel functional regulator III in children with class III malocclusions. American Journal of Orthodontics & Dentofacial Orthopedics 125(3): 294–301

31. Baram D, Yang Y, Ren C et al. (2019) Orthodontic Treatment Need and the Psychosocial Impact of Malocclusion in 12-Year-Old Hong Kong Children. Thescientificworldjournal 2019: 2685437. doi: 10.1155/2019/2685437

32. Barrett AA, Baccetti T, McNamara,JA,Jr (2010) Treatment effects of the light-force chincup. American Journal of Orthodontics & Dentofacial Orthopedics 138(4): 468–476. doi: 10.1016/j.ajodo.2008.12.024

33. Basciftci FA, Uysal T, Buyukerkmen A et al. (2003) The effects of activator treatment on the craniofacial structures of Class II division 1 patients. European Journal of Orthodontics 25(1): 87–93

34. Bassarelli T, Franchi L, Defraia E et al. (2016) Dentoskeletal effects produced by a Jasper Jumper with an anterior bite plane. Angle Orthodontist 86(5): 775–781. doi: 10.2319/110115-737.1

35. Bates CJ, McDonald JP (2005) The relationship between severity of obstructive sleep apnoea/hypopnoea syndrome (OSAHS) and lateral cephalometric radiograph values: a clinical diagnostic tool. Surg. J. R. Coll. Surg. Edinb. Irel. 3(5): 338–346. doi: 10.1016/s1479-666x(05)80113-1

36. Batista KB, Thiruvenkatachari B, Harrison JE et al. (2018) Orthodontic treatment for prominent upper front teeth (Class II malocclusion) in children and adolescents. [Review]. Cochrane Database of Systematic Reviews 3: CD003452. doi: 10.1002/14651858.CD003452.pub4

37. Battagel JM, Orton HS (1991) Class III malocclusion: a comparison of extraction and non-extraction techniques. European Journal of Orthodontics 13(3): 212–222

38. Battagel JM, Orton HS (1995) A comparative study of the effects of customized facemask therapy or headgear to the lower arch on the developing Class III face. European Journal of Orthodontics 17(6): 467–482

39. Bauss O, Freitag S, Röhling J et al. (2008) Influence of overjet and lip coverage on the prevalence and severity of incisor trauma. J. Orofac. Orthop. 69(6): 402–410. doi: 10.1007/s00056-008-8805-1

40. Bavbek NC, Tuncer BB, Turkoz C et al. (2016) Changes in airway dimensions and hyoid bone position following class II correction with forsus fatigue resistant device. Clin. Oral Investig. 20(7): 1747–1755. doi: 10.1007/s00784-015-1659-1

41. Baysal A, Ozturk MA, Sahan AO et al. (2016) Facial soft-tissue changes after rapid maxillary expansion analyzed with 3-dimensional stereophotogrammetry: A randomized, controlled clinical trial. Angle Orthodontist 86(6): 934–942

42. Bernabe E, Sheiham A, Tsakos G et al. (2008) The impact of orthodontic treatment on the quality of life in adolescents: a case-control study. European Journal of Orthodontics 30(5): 515–520. doi: 10.1093/ejo/cjn026

43. Bicakci AA, Agar U, Sokucu O et al. (2005) Nasal airway changes due to rapid maxillary expansion timing. Angle Orthodontist 75(1): 1–6

44. Bilbo EE, Marshall SD, Southard KA et al. (2018) Long-term skeletal effects of high-pull headgear followed by fixed appliances for the treatment of Class II malocclusions. Angle Orthodontist 88(5): 530–537. doi: 10.2319/091517-620.1

45. Borzabadi-Farahani A, Borzabadi-Farahani A (2011) The association between orthodontic treatment need and maxillary incisor trauma, a retrospective clinical study. Oral Surg. Oral Med. Oral Pathol. Oral Radiol. Endod. 112(6): e75-80. doi: 10.1016/j.tripleo.2011.05.024

46. Borzabadi-Farahani A, Lane CJ, Yen SL (2014) Late maxillary protraction in patients with unilateral cleft lip and palate: a retrospective study. Cleft Palate Craniofacial Journal 51(1): e1-e10. doi: 10.1597/12-099

47. Bourdiol P, Soulier-Peigue D, Lachaze P et al. (2017) Only severe malocclusion correlates with mastication deficiency. Arch. Oral Biol. 75: 14–20. doi: 10.1016/j.archoralbio.2016.12.002

48. Bucci R, Montanaro D, Rongo R et al. (2019) Effects of maxillary expansion on the upper airways: Evidence from systematic reviews and meta-analyses. [Review]. Journal of Oral Rehabilitation 46(4): 377–387. doi: 10.1111/joor.12766

49. Burden DJ (1995) An investigation of the association between overjet size, lip coverage, and traumatic injury to maxillary incisors. Eur. J. Orthodont. 17(6): 513–517. doi: 10.1093/ejo/17.6.513

50. Buyuknacar GB, Gulec A (2020) Correlation between the cephalometric measurements and acoustic properties of /s/ sound in Turkish. J. Appl. Oral Sci. 28: e20190399. doi: 10.1590/1678-7757-2019-0399

51. Cacciatore G, Ghislanzoni LT, Alvetro L et al. (2014) Treatment and posttreatment effects induced by the Forsus appliance: A controlled clinical study. Angle Orthodontist 84(6): 1010–1017. doi: 10.2319/112613-867.1

52. Chadwick SM, Aird JC, Taylor PJ et al. (2001) Functional regulator treatment of Class II division 1 malocclusions. Eur. J. Orthodont. 23(5): 495–505. doi: 10.1093/ejo/23.5.495

53. Chatzoudi MI, Ioannidou-Marathiotou I, Papadopoulos MA (2014) Clinical effectiveness of chin cup treatment for the management of Class III malocclusion in pre-pubertal patients: a systematic review and meta-analysis. [Review]. Progress in Orthodontics 15: 62. doi: 10.1186/s40510-014-0062-9

54. Chen DR, McGorray SP, Dolce C et al. (2011) Effect of early Class II treatment on the incidence of incisor trauma. American Journal of Orthodontics & Dentofacial Orthopedics 140(4): e155-60. doi: 10.1016/j.ajodo.2011.02.023

55. Chen X, Liu D, Liu J et al. (2015) Three-Dimensional Evaluation of the Upper Airway Morphological Changes in Growing Patients with Skeletal Class III Malocclusion

Treated by Protraction Headgear and Rapid Palatal Expansion: A Comparative Research. PLoS ONE [Electronic Resource] 10(8): e0135273. doi: 10.1371/journal.pone.0135273

56. Chiqueto K, Henriques JF, Barros SE et al. (2013) Angle Class II correction with MARA appliance. Dental Press Journal of Orthodontics 18(1): 35–44

57. Choi T-H, Kim B-I, Chung CJ et al. (2015) Assessment of masticatory function in patients with non-sagittal occlusal discrepancies. J. Oral Rehabil. 42(1): 2–9. doi: 10.1111/joor.12227

58. Cochrane Deutschland Stiftung, Institut für Evidenz in der Medizin, Institut für Medizinische Biometrie und Statistik, Freiburg et al. (2019) Manual systematische Recherche für Evidenzsynthesen und Leitlinien, Version 2.0

59. Cordasco G, Matarese G, Rustico L et al. (2014) Efficacy of orthopedic treatment with protraction facemask on skeletal Class III malocclusion: a systematic review and meta-analysis. [Review]. Orthodontics & Craniofacial Research 17(3): 133–143. doi: 10.1111/ocr.12040

60. Corrêa-Faria P, Martins CC, Bönecker M et al. (2016) Clinical factors and socio-demographic characteristics associated with dental trauma in children: a systematic review and meta-analysis. Dent Traumatol 32(5): 367–378. doi: 10.1111/edt.12268

61. Cozza P, Baccetti T, Mucedero M et al. (2010) Treatment and posttreatment effects of a facial mask combined with a bite-block appliance in Class III malocclusion. American Journal of Orthodontics & Dentofacial Orthopedics 138(3): 300–310. doi: 10.1016/j.ajodo.2010.05.001

62. Cozza P, De,Toffol,L, Colagrossi S (2004) Dentoskeletal effects and facial profile changes during activator therapy. European Journal of Orthodontics 26(3): 293–302

63. Croft RS, Buschang PH, English JD et al. (1999) A cephalometric and tomographic evaluation of Herbst treatment in the mixed dentition. American Journal of Orthodontics & Dentofacial Orthopedics 116(4): 435–443

64. Danaei SM, Ajami S, Etemadi H et al. (2018) Assessment of the effect of maxillary protraction appliance on pharyngeal airway dimensions in relation to changes in tongue posture. Dental Research Journal 15(3): 208–214

65. de,Almeida,MR, Flores-Mir C, Brandao AG et al. (2008) Soft tissue changes produced by a banded-type Herbst appliance in late mixed dentition patients. World Journal of Orthodontics 9(2): 121–131

66. De,Clerck,H, Cevidanes L, Baccetti T (2010) Dentofacial effects of bone-anchored maxillary protraction: a controlled study of consecutively treated Class III patients. American Journal of Orthodontics & Dentofacial Orthopedics 138(5): 577–581. doi: 10.1016/j.ajodo.2009.10.037

67. de,Oliveira,CM, Sheiham A (2004) Orthodontic treatment and its impact on oral health-related quality of life in Brazilian adolescents. Journal of Orthodontics 31(1): 20-7; discussion 15

68. Defraia E, Marinelli A, Baroni G et al. (2008) Dentoskeletal effects of a removable appliance for expansion of the maxillary arch: a postero-anterior cephalometric study. European Journal of Orthodontics 30(1): 57–60

69. Deguchi T, McNamara JA (1999) Craniofacial adaptations induced by chincup therapy in Class III patients. American Journal of Orthodontics & Dentofacial Orthopedics 115(2): 175–182

70. Dimberg L, Arnrup K, Bondemark L (2015) The impact of malocclusion on the quality of life among children and adolescents: a systematic review of quantitative studies. [Review]. European Journal of Orthodontics 37(3): 238–247. doi: 10.1093/ejo/cju046

71. Dogan S (2012) The effects of face mask therapy in cleft lip and palate patients. Annals of Maxillofacial Surgery 2(2): 116–120. doi: 10.4103/2231-0746.101332

72. Dorri M (2015) In children with prominent lower front teeth (class III malocclusion), how does orthodontic treatment affect outcomes? Cochrane Clinical Answers. doi: 10.1002/cca.996

73. Doshi UH, Bhad-Patil WA (2011) Speech defect and orthodontics: a contemporary review. Orthodontics : The Art and Practice of Dentofacial Enhancement 12(4): 340–353

74. Duarte-Rodrigues L, Ramos-Jorge ML, Alves-Duarte AC et al. (2020) Oral disorders associated with the experience of verbal bullying among Brazilian school-aged children: A case-control study. Journal of the American Dental Association 151(6): 399–406. doi: 10.1016/j.adaj.2020.02.001

75. Ehmer U, Tulloch CJ, Proffit WR et al. (1999) An international comparison of early treatment of angle Class-II/1 cases. Skeletal effects of the first phase of a prospective clinical trial. Journal of Orofacial Orthopedics 60(6): 392–408

76. Eissa O, ElShennawy M, Gaballah S et al. (2018) Treatment of Class III malocclusion using miniscrew-anchored inverted Forsus FRD: Controlled clinical trial. Angle Orthodontist 88(6): 692–701. doi: 10.2319/110717-760.1

77. Eissa O, El-Shennawy M, Gaballah S et al. (2017) Treatment outcomes of Class II malocclusion cases treated with miniscrew-anchored Forsus Fatigue Resistant Device: A randomized controlled trial. Angle Orthodontist 87(6): 824–833. doi: 10.2319/032717-214.1

78. Elfeky HY, Fayed MS, AlHammadi MS et al. (2018) Three-dimensional skeletal, dentoalveolar and temporomandibular joint changes produced by Twin Block functional appliance. J. Orofac. Orthop. 79(4): 245–258. doi: 10.1007/s00056-018-0137-1

79. ElKordy SA, Abouelezz AM, Fayed MM et al. (2016) Three-dimensional effects of the mini-implant-anchored Forsus Fatigue Resistant Device: A randomized controlled trial. Angle Orthodontist 86(2): 292–305. doi: 10.2319/012515-55.1

80. ElKordy SA, Abouelezz AM, Fayed MM et al. (2019) Evaluation of the miniplate-anchored Forsus Fatigue Resistant Device in skeletal Class II growing subjects: A randomized controlled trial. Angle Orthodontist 89(3): 391–403. doi: 10.2319/062018-468.1

81. English JD, Buschang PH, Throckmorton GS (2002) Does malocclusion affect masticatory performance? Angle Orthod. 72(1): 21–27. doi: 10.1043/0003-3219(2002)072<0021:DMAMP>2.0.CO;2

82. Entrenas I, Gonzalez-Chamorro E, Alvarez-Abad C et al. (2019) Evaluation of changes in the upper airway after Twin Block treatment in patients with Class II malocclusion. Clinical & Experimental Dental Research 5(3): 259–268. doi: 10.1002/cre2.180

83. Erdinc AE, Ugur T, Erbay E (1999) A comparison of different treatment techniques for posterior crossbite in the mixed dentition. Am. J. Orthod. Dentofac. Orthop. 116(3): 287–300. doi: 10.1016/S0889-5406(99)70240-4

84. Faco R, Yatabe M, Cevidanes LH et al. (2019) Bone-anchored maxillary protraction in unilateral cleft lip and palate: a cephalometric appraisal. European Journal of Orthodontics 019. doi: 10.1093/ejo/cjz005

85. Falck F, Zimmermann-Menzel K (2008) Cephalometric changes in the treatment of Class III using the Frankel appliance. J. Orofac. Orthop. 69(2): 99–109. doi: 10.1007/s00056-008-0716-7

86. Faltin KJ, Faltin RM, Baccetti T et al. (2003) Long-term effectiveness and treatment timing for Bionator therapy. Angle Orthodontist 73(3): 221–230

87. Farronato G, Giannini L, Riva R et al. (2012) Correlations between malocclusions and dyslalias. European Journal of Paediatric Dentistry 13(1): 13–18

88. Feldens CA, Borges TS, Vargas-Ferreira F et al. (2016) Risk factors for traumatic dental injuries in the primary dentition: concepts, interpretation, and evidence. Dental Traumatology 32(6): 429–437. doi: 10.1111/edt.12281

89. Feng X, Li J, Li Y et al. (2012) Effectiveness of TAD-anchored maxillary protraction in late mixed dentition A systematic review. Angle Orthod. 82(6): 1107–1114. doi: 10.2319/111411-705.1

90. Ferrando-Magraner E, Garcia-Sanz V, Bellot-Arcis C et al. (2019) Oral health-related quality of life of adolescents after orthodontic treatment. A systematic review. [Review]. Journal of Clinical & Experimental Dentistry 11(2): e194-e202. doi: 10.4317/jced.55527

91. Firouz M, Zernik J, Nanda R (1992) Dental and orthopedic effects of high-pull headgear in treatment of Class II, division 1 malocclusion. American Journal of Orthodontics & Dentofacial Orthopedics 102(3): 197–205

92. Flores-Mir C, Ayeh A, Goswani A et al. (2007) Skeletal and dental changes in Class II division 1 malocclusions treated with splint-type Herbst appliances. A systematic review. [Review] [36 refs]. Angle Orthodontist 77(2): 376–381

93. Flores-Mir C, Barnett G, Higgins DW et al. (2009) Short-term skeletal and dental effects of the Xbow appliance as measured on lateral cephalograms. American Journal of Orthodontics & Dentofacial Orthopedics 136(6): 822–832. doi: 10.1016/j.ajodo.2008.01.021

94. Flores-Mir C, Major MP, Major PW (2006) Soft tissue changes with fixed functional appliances in Class II division 1. [Review] [46 refs]. Angle Orthodontist 76(4): 712–720

95. Flores-Mir C, Major PW (2006) Cephalometric facial soft tissue changes with the twin block appliance in Class II division 1 malocclusion patients. A systematic review. [Review] [39 refs]. Angle Orthodontist 76(5): 876–881

96. Franchi L, Alvetro L, Giuntini V et al. (2011) Effectiveness of comprehensive fixed appliance treatment used with the Forsus Fatigue Resistant Device in Class II patients. Angle Orthodontist 81(4): 678–683. doi: 10.2319/102710-629.1

97. Franchi L, Baccetti T, Cameron CG et al. (2002) Thin-plate spline analysis of the short- and long-term effects of rapid maxillary expansion. Eur. J. Orthodont. 24(2): 143–150. doi: 10.1093/ejo/24.2.143

98. Franchi L, Baccetti T, McNamara JA (2004) Postpubertal assessment of treatment timing for maxillary expansion and protraction therapy followed by fixed appliances. American Journal of Orthodontics & Dentofacial Orthopedics 126(5): 555–568

99. Franchi L, Baccetti T, McNamara,JA,Jr (1998) Shape-coordinate analysis of skeletal changes induced by rapid maxillary expansion and facial mask therapy. American Journal of Orthodontics & Dentofacial Orthopedics 114(4): 418–426

100. Franchi L, Pavoni C, Faltin,K,Jr et al. (2013) Long-term skeletal and dental effects and treatment timing for functional appliances in Class II malocclusion. Angle Orthodontist 83(2): 334–340. doi: 10.2319/052912-450.1

101. Freeman DC, McNamara,JA,Jr, Baccetti T et al. (2009) Long-term treatment effects of the FR-2 appliance of Frankel. American Journal of Orthodontics & Dentofacial Orthopedics 135(5): 570.e1-6; discussion 570-1. doi: 10.1016/j.ajodo.2007.11.029

102. Gameiro GH, Magalhaes IB, Szymanski MM et al. (2017) Is the main goal of mastication achieved after orthodontic treatment? A prospective longitudinal study. Dental Press Journal of Orthodontics 22(3): 72–78. doi: 10.1590/2177-6709.22.3.072-078.oar

103. Garattini G, Levrini L, Crozzoli P et al. (1998) Skeletal and dental modifications produced by the Bionator III appliance. Am. J. Orthod. Dentofac. Orthop. 114(1): 40–44. doi: 10.1016/S0889-5406(98)70235-5

104. Gencer D, Kaygisiz E, Yuksel S et al. (2015) Comparison of double-plate appliance/facemask combination and facemask therapy in treating class III malocclusions. Angle Orthodontist 85(2): 278–283. doi: 10.2319/013114-83.1

105. Geran RG, McNamara,JA,Jr, Baccetti T et al. (2006) A prospective long-term study on the effects of rapid maxillary expansion in the early mixed dentition. American Journal of Orthodontics & Dentofacial Orthopedics 129(5): 631–640

106. Ghislanzoni LT, Baccetti T, Toll D et al. (2013) Treatment timing of MARA and fixed appliance therapy of Class II malocclusion. European Journal of Orthodontics 35(3): 394–400. doi: 10.1093/ejo/cjs023

107. Ghislanzoni LT, Toll DE, Defraia E et al. (2011) Treatment and posttreatment outcomes induced by the Mandibular Advancement Repositioning Appliance; a controlled clinical study. Angle Orthodontist 81(4): 684–691. doi: 10.2319/111010-656.1

108. Giuntini V, Vangelisti A, Masucci C et al. (2015) Treatment effects produced by the Twin-block appliance vs the Forsus Fatigue Resistant Device in growing Class II patients. Angle Orthodontist 85(5): 784–789. doi: 10.2319/090514-624.1

109. Glasl B, Ludwig B, Schopf P (2006) Prevalence and development of KIG-relevant symptoms in primary school students from Frankfurt am Main. Journal of Orofacial Orthopedics 67(6): 414–423

110. Godoy F, Godoy-Bezerra J, Rosenblatt A (2011) Treatment of posterior crossbite comparing 2 appliances: a community-based trial. American Journal of Orthodontics & Dentofacial Orthopedics 139(1): e45-52. doi: 10.1016/j.ajodo.2010.06.017

111. Goyenc Y, Ersoy S (2004) The effect of a modified reverse headgear force applied with a facebow on the dentofacial structures. European Journal of Orthodontics 26(1): 51–57

112. Guest SS, McNamara,JA,Jr, Baccetti T et al. (2010) Improving Class II malocclusion as a side-effect of rapid maxillary expansion: a prospective clinical study. American Journal of Orthodontics & Dentofacial Orthopedics 138(5): 582–591. doi: 10.1016/j.ajodo.2008.12.026

113. Guimaraes,CH,Jr, Henriques JF, Janson G et al. (2013) Prospective study of dentoskeletal changes in Class II division malocclusion treatment with twin force bite corrector. Angle Orthodontist 83(2): 319–326. doi: 10.2319/042312-339.1

114. Hanoun A, Al-Jewair TS, Tabbaa S et al. (2014) A comparison of the treatment effects of the Forsus Fatigue Resistance Device and the Twin Block appliance in patients with class II malocclusions. Clinical Cosmetic & Investigational Dentistry 6: 57–63. doi: 10.2147/CCIDE.S64119

115. Hansson C, Skold B, Linder-Aronson S (1997) Treatment of adolescents with Hansaplate/headgear. Influence on face in profile and on dentition. Journal of Orofacial Orthopedics 58(1): 16–29

116. Harrison JE, Ashby D (2001) Orthodontic treatment for posterior crossbites. [Review] [43 refs]. Cochrane Database of Systematic Reviews(1): CD000979

117. Hassan AH, Amin H-S (2010) Association of orthodontic treatment needs and oral health-related quality of life in young adults. American Journal of Orthodontics & Dentofacial Orthopedics 137(1): 42–47. doi: 10.1016/j.ajodo.2008.02.024

118. Heinrichs DA, Shammaa I, Martin C et al. (2014) Treatment effects of a fixed intermaxillary device to correct class II malocclusions in growing patients. Progress in Orthodontics 15: 45. doi: 10.1186/s40510-014-0045-x

119. Helm S, Kreiborg S, Solow B (1985) Psychosocial implications of malocclusion: a 15-year follow-up study in 30-year-old Danes. American Journal of Orthodontics 87(2): 110–118. doi: 10.1016/0002-9416(85)90020-x

120. Huynh NT, Morton PD, Rompré PH et al. (2011) Associations between sleep-disordered breathing symptoms and facial and dental morphometry, assessed with screening examinations. American Journal of Orthodontics & Dentofacial Orthopedics 140(6): 762–770. doi: 10.1016/j.ajodo.2011.03.023

121. Iwasaki T, Takemoto Y, Inada E et al. (2014) Three-dimensional cone-beam computed tomography analysis of enlargement of the pharyngeal airway by the Herbst appliance. Am. J. Orthod. Dentofac. Orthop. 146(6): 776–785. doi: 10.1016/j.ajodo.2014.08.017

122. Jakobsone G, Latkauskiene D, McNamara Jr JA (2013) Mechanisms of Class II correction induced by the crown Herbst appliance as a single-phase Class II therapy: 1 year follow-up. Progress in Orthodontics 14(1). doi: 10.1186/2196-1042-14-27

123. Jamilian A, Kiaee B, Sanayei S et al. (2016) Orthodontic Treatment of Malocclusion and its Impact on Oral Health-Related Quality of Life. The open dentistry journal 10: 236–241. doi: 10.2174/1874210601610010236

124. Janson GR, da,Silva,CC, Bergersen EO et al. (2000) Eruption Guidance Appliance effects in the treatment of Class II, Division 1 malocclusions. American Journal of Orthodontics & Dentofacial Orthopedics 117(2): 119–129

125. Janson GR, Toruno JL, Martins DR et al. (2003) Class II treatment effects of the Frankel appliance. European Journal of Orthodontics 25(3): 301–309

126. Järvinen S (1978) Incisal overjet and traumatic injuries to upper permanent incisors. A retrospective study. Acta Odontologica Scandinavica 36(6): 359–362. doi: 10.3109/00016357809029088

127. Järvinen S (1979) Traumatic injuries to upper permanent incisors related to age and incisal overjet. A retrospective study. Acta Odontologica Scandinavica 37(6): 335–338. doi: 10.3109/00016357909004705

128. Javidi H, Vettore M, Benson PE (2017) Does orthodontic treatment before the age of 18 years improve oral health-related quality of life? A systematic review and meta-analysis. Am. J. Orthod. Dentofac. Orthop. 151(4): 644–655. doi: 10.1016/j.ajodo.2016.12.011

129. Jena AK, Duggal R (2010) Treatment Effects of Twin-Block and Mandibular Protraction Appliance-IV in the Correction of Class II Malocclusion. Angle Orthod. 80(3): 485–491. doi: 10.2319/062709-359.1

130. Jena AK, Duggal R, Parkash H (2006) Skeletal and dentoalveolar effects of Twin-block and bionator appliances in the treatment of Class II malocclusion: a comparative study. American Journal of Orthodontics & Dentofacial Orthopedics 130(5): 594–602

131. Johnson NC, Sandy JR (1999) Tooth position and speech--is there a relationship? Angle Orthodontist 69(4): 306–310. doi: 10.1043/0003-3219(1999)069<0306:TPASIT>2.3.CO;2

132. Julku J, Pirila-Parkkinen K, Pirttiniemi P (2018) Airway and hard tissue dimensions in children treated with early and later timed cervical headgear-a randomized controlled trial. European Journal of Orthodontics 40(3): 285–295. doi: 10.1093/ejo/cjx088

133. Jung M-H (2010) Evaluation of the effects of malocclusion and orthodontic treatment on self-esteem in an adolescent population. American Journal of Orthodontics & Dentofacial Orthopedics 138(2): 160–166. doi: 10.1016/j.ajodo.2008.08.040

134. Kajiyama K, Murakami T, Suzuki A (2000) Evaluation of the modified maxillary protractor applied to Class III malocclusion with retruded maxilla in early mixed dentition. American Journal of Orthodontics & Dentofacial Orthopedics 118(5): 549–559

135. Kajiyama K, Murakami T, Suzuki A (2004) Comparison of orthodontic and orthopedic effects of a modified maxillary protractor between deciduous and early mixed dentitions. American Journal of Orthodontics & Dentofacial Orthopedics 126(1): 23–32

136. Kaluza S, Hiller K-A, Proff P et al. (2019) Draft search strategies for "Relapse in orthognatic surgery: a systematic review and meta-analysis". Universität Regensburg

137. Kama JD, Ozer T, Baran S (2006) Orthodontic and orthopaedic changes associated with treatment in subjects with Class III malocclusions. European Journal of Orthodontics 28(5): 496–502

138. Kamal AT, Fida M (2019) Evaluation of cervical spine posture after functional therapy with twin-block appliances: A retrospective cohort study. Am. J. Orthod. Dentofac. Orthop. 155(5): 656–661. doi: 10.1016/j.ajodo.2018.06.012

139. Kang Y, Franchi L, Manton DJ et al. (2018) A cephalometric study of the skeletal and dento-alveolar effects of the modified Louisiana State University activator in Class II malocclusion. Eur. J. Orthodont. 40(2): 164–175. doi: 10.1093/ejo/cjx044

140. Kania MJ, Keeling SD, McGorray SP et al. (1996) Risk factors associated with incisor injury in elementary school children. Angle Orthod. 66(6): 423–432. doi: 10.1043/0003-3219(1996)066<0423:RFAWII>2.3.CO;2

141. Khosravanifard B, Ghanbari-Azarnir S, Rakhshan H et al. (2012) Association between orthodontic treatment need and masticatory performance. Orthodontics : The Art and Practice of Dentofacial Enhancement 13(1): e20-8

142. Kilinc AS, Arslan SG, Kama JD et al. (2008) Effects on the sagittal pharyngeal dimensions of protraction and rapid palatal expansion in Class III malocclusion subjects. European Journal of Orthodontics 30(1): 61–66

143. Kim Y-J, Hong J-S, Hwang Y-I et al. (2010) Three-dimensional analysis of pharyngeal airway in preadolescent children with different anteroposterior skeletal patterns. American Journal of Orthodontics & Dentofacial Orthopedics 137(3): 306.e1-11; discussion 306-7. doi: 10.1016/j.ajodo.2009.10.025

144. Kim-Berman H, McNamara,JA,Jr, Lints JP et al. (2019) Treatment effects of the Carriere Motion 3DTM appliance for the correction of Class II malocclusion in adolescents. Angle Orthodontist 019. doi: 10.2319/121418-872.1

145. Kobayashi T, Honma K, Nakajima T et al. (1993) Masticatory function in patients with mandibular prognathism before and after orthognathic surgery. Journal of Oral & Maxillofacial Surgery 51(9): 997-1001; discussion 1002-3. doi: 10.1016/s0278-2391(10)80043-6

146. Kobayashi T, Honma K, Shingaki S et al. (2001) Changes in masticatory function after orthognathic treatment in patients with mandibular prognathism. Br. J. Oral Maxillofac. Surg. 39(4): 260–265. doi: 10.1054/bjom.2000.0576

147. Koike S, Sujino T, Ohmori H et al. (2013) Gastric emptying rate in subjects with malocclusion examined by (13) C breath test. J. Oral Rehabil. 40(8): 574–581. doi: 10.1111/joor.12073

148. Kragt L, Dhamo B, Wolvius EB et al. (2016) The impact of malocclusions on oral health-related quality of life in children-a systematic review and meta-analysis. Clin. Oral Investig. 20(8): 1881–1894. doi: 10.1007/s00784-015-1681-3

149. Kragt L, Jaddoe V, Wolvius E et al. (2017) The association of subjective orthodontic treatment need with oral health-related quality of life. Community Dentist. Oral Epidemiol. 45(4): 365–371. doi: 10.1111/cdoe.12299

150. Kucukkeles N, Ilhan I, Orgun IA (2007) Treatment efficiency in skeletal Class II patients treated with the jasper jumper. Angle Orthodontist 77(3): 449–456

151. Kunz F, Platte P, Keß S et al. (2018) Correlation between oral health-related quality of life and orthodontic treatment need in children and adolescents-a prospective interdisciplinary multicentre cohort study (Zusammenhang zwischen mundgesundheitsbezogener Lebensqualität und kieferorthopädischem Behandlungsbedarf bei Kindern und Jugendlichen – Eine prospektive, interdisziplinäre und multizentrische Kohortenstudie). Journal of Orofacial Orthopedics 79(5): 297–308. doi: 10.1007/s00056-018-0142-4

152. Kunz F, Platte P, Keß S et al. (2019) Auswirkungen spezifischer kieferorthopädischer Parameter auf die mundgesundheitsbezogene Lebensqualität von Kindern und Jugendlichen : Eine prospektive, interdisziplinäre und multizentrische Kohortenstudie (Impact of specific orthodontic parameters on the oral health-related quality of life in children and adolescents : A prospective interdisciplinary, multicentre, cohort study). J Orofac Orthop 80(4): 174–183. doi: 10.1007/s00056-019-00181-x

153. LaHaye MB, Buschang PH, Alexander RG et al. (2006) Orthodontic treatment changes of chin position in Class II Division 1 patients. American Journal of Orthodontics & Dentofacial Orthopedics 130(6): 732–741

154. Lange DW, Kalra V, Broadbent,BH,Jr et al. (1995) Changes in soft tissue profile following treatment with the bionator. Angle Orthodontist 65(6): 423–430

155. Laranjo F, Pinho T (2014) Cephalometric study of the upper airways and dentoalveolar height in open bite patients. International Orthodontics 12(4): 467–482. doi: 10.1016/j.ortho.2014.10.005

156. Latkauskiene D, Jakobsone G (2012) Immediate post-treatment crowned Herbst effects in growing patients. Stomatologija 14(3): 89–92

157. Lee D-H, Yu H-S (2012) Masseter muscle changes following orthognathic surgery A long-term three-dimensional computed tomography follow-up. Angle Orthod. 82(5): 792–798. doi: 10.2319/111911-717.1

158. Lee W-C, Tu Y-K, Huang C-S et al. (2018) Pharyngeal airway changes following maxillary expansion or protraction: A meta-analysis. Orthod. Craniofac. Res. 21(1): 4–11. doi: 10.1111/ocr.12208

159. Levin AS, McNamara,JA,Jr, Franchi L et al. (2008) Short-term and long-term treatment outcomes with the FR-3 appliance of Frankel. American Journal of Orthodontics & Dentofacial Orthopedics 134(4): 513–524. doi: 10.1016/j.ajodo.2006.10.036

160. Li L, Liu H, Cheng H et al. (2014) CBCT evaluation of the upper airway morphological changes in growing patients of class II division 1 malocclusion with mandibular retrusion using twin block appliance: a comparative research. PLoS ONE [Electronic Resource] 9(4): e94378. doi: 10.1371/journal.pone.0094378

161. Lin HC, Chang HP, Chang HF (2007) Treatment effects of occipitomental anchorage appliance of maxillary protraction combined with chincup traction in children with Class III malocclusion. Journal of the Formosan Medical Association 106(5): 380–391

162. Lippold C, Stamm T, Meyer U et al. (2013) Early treatment of posterior crossbite--a randomised clinical trial. Trials [Electronic Resource] 14: 20. doi: 10.1186/1745-6215-14-20

163. Lisson JA, Mokrys K, Kinzinger GS et al. (2013) Changes in soft-tissue profiles after treatment of class II/1 patients with bite-jumping appliances. Journal of Orofacial Orthopedics 74(2): 113–123. doi: 10.1007/s00056-012-0128-6

164. Liu Z, McGrath C, Hägg U (2009) The impact of malocclusion/orthodontic treatment need on the quality of life. A systematic review. Angle Orthodontist 79(3): 585–591. doi: 10.2319/042108-224.1

165. Liu ZP, Li CJ, Hu HK et al. (2011) Efficacy of short-term chincup therapy for mandibular growth retardation in Class III malocclusion. [Review]. Angle Orthodontist 81(1): 162–168. doi: 10.2319/050510-244.1

166. Lombardo G, Vena F, Negri P et al. (2020) Worldwide prevalence of malocclusion in the different stages of dentition: A systematic review and meta-analysis. Eur. J. Paediatr. Dent. 21(2): 115–122. doi: 10.23804/ejpd.2020.21.02.05

167. Lucchese A, Carinci F, Brunelli G (2012) SKELETAL EFFECTS INDUCED BY TWIN BLOCK IN THERAPY OF CLASS II MALOCCLUSION. Eur. J. Inflamm. 10(1): 83–87

168. Lund DI, Sandler PJ (1998) The effects of Twin Blocks: a prospective controlled study. American Journal of Orthodontics & Dentofacial Orthopedics 113(1): 104–110

169. Lux CJ, Ducker B, Pritsch M et al. (2009) Occlusal status and prevalence of occlusal malocclusion traits among 9-year-old schoolchildren. Eur. J. Orthodont. 31(3): 294–299. doi: 10.1093/ejo/cjn116

170. Lux CJ, Rubel J, Starke J et al. (2001) Effects of early activator treatment in patients with class II malocclusion evaluated by thin-plate spline analysis. Angle Orthodontist 71(2): 120–126

171. Macdonald KE, Kapust AJ, Turley PK (1999) Cephalometric changes after the correction of Class III malocclusion with maxillary expansion facemask therapy. Am. J. Orthod. Dentofac. Orthop. 116(1): 13–24. doi: 10.1016/S0889-5406(99)70298-2

172. Magalhães IB, Pereira LJ, Marques LS et al. (2010) The influence of malocclusion on masticatory performance. A systematic review. Angle Orthodontist 80(5): 981–987. doi: 10.2319/011910-33.1

173. Magno MB, Nadelman P, Leite, Karla Lorene de França et al. (2020) Associations and risk factors for dental trauma: A systematic review of systematic reviews. Community Dentist. Oral Epidemiol. doi: 10.1111/cdoe.12574

174. Malta LA, Baccetti T, Franchi L et al. (2010) Long-term dentoskeletal effects and facial profile changes induced by bionator therapy. Angle Orthodontist 80(1): 10–17. doi: 10.2319/031609-156.1

175. Mandall N, Cousley R, DiBiase A et al. (2016) Early class III protraction facemask treatment reduces the need for orthognathic surgery: a multi-centre, two-arm parallel randomized, controlled trial. Journal of Orthodontics 43(3): 164–175. doi: 10.1080/14653125.2016.1201302

176. Mandall N, DiBiase A, Littlewood S et al. (2010) Is early Class III protraction facemask treatment effective? A multicentre, randomized, controlled trial: 15-month follow-up. Journal of Orthodontics 37(3): 149–161. doi: 10.1179/14653121043056

177. Mandall NA, McCord JF, Blinkhorn AS et al. (2000) Perceived aesthetic impact of malocclusion and oral self-perceptions in 14-15-year-old Asian and Caucasian children in greater Manchester. European Journal of Orthodontics 22(2): 175–183

178. Mantysaari R, Kantomaa T, Pirttiniemi P et al. (2004) The effects of early headgear treatment on dental arches and craniofacial morphology: a report of a 2 year randomized study. European Journal of Orthodontics 26(1): 59–64

179. Marquezin MCS, Kobayashi FY, Montes ABM et al. (2013) Assessment of masticatory performance, bite force, orthodontic treatment need and orofacial dysfunction in children and adolescents. Arch. Oral Biol. 58(3): 286–292. doi: 10.1016/j.archoralbio.2012.06.018

180. Marsico E, Gatto E, Burrascano M et al. (2011) Effectiveness of orthodontic treatment with functional appliances on mandibular growth in the short term. [Review]. American Journal of Orthodontics & Dentofacial Orthopedics 139(1): 24–36. doi: 10.1016/j.ajodo.2010.04.028

181. Martina R, Cioffi I, Galeotti A et al. (2013) Efficacy of the Sander bite-jumping appliance in growing patients with mandibular retrusion: a randomized controlled trial. Orthodontics & Craniofacial Research 16(2): 116–126. doi: 10.1111/ocr.12013

182. Martina R, D'Anto V, De,Simone,V et al. (2019) Cephalometric outcomes of a new orthopaedic appliance for Class III malocclusion treatment. European Journal of Orthodontics 019. doi: 10.1093/ejo/cjz037

183. Masood M, Masood Y, Newton T (2014) Cross-bite and oral health related quality of life in young people. J. Dent. 42(3): 249–255. doi: 10.1016/j.jdent.2013.12.004

184. Maspero C, Giannini L, Galbiati G et al. (2015) Upper airway obstuction in class II patients. Effects of Andresen activator on the anatomy of pharingeal airway passage. Cone beam evalution. Stomatologija 17(4): 124–130

185. Maspero C, Prevedello C, Giannini L et al. (2014) Atypical swallowing: a review. Minerva Stomatologica 63(6): 217–227

186. Masucci C, Franchi L, Giuntini V et al. (2014) Short-term effects of a modified Alt-RAMEC protocol for early treatment of Class III malocclusion: a controlled study. Orthodontics & Craniofacial Research 17(4): 259–269. doi: 10.1111/ocr.12051

187. McNamara,JA,Jr, Sigler LM, Franchi L et al. (2010) Changes in occlusal relationships in mixed dentition patients treated with rapid maxillary expansion. A prospective clinical study. Angle Orthodontist 80(2): 230–238. doi: 10.2319/040309-192.1

188. Merwin D, Ngan P, Hagg U et al. (1997) Timing for effective application of anteriorly directed orthopedic force to the maxilla. American Journal of Orthodontics & Dentofacial Orthopedics 112(3): 292–299

189. Millett DT, Cunningham SJ, O'Brien KD et al. (2018) Orthodontic treatment for deep bite and retroclined upper front teeth in children. [Review]. Cochrane Database of Systematic Reviews 2: CD005972. doi: 10.1002/14651858.CD005972.pub4

190. Mills CM, McCulloch KJ (1998) Treatment effects of the twin block appliance: A cephalometric study. Am. J. Orthod. Dentofac. Orthop. 114(1): 15–24. doi: 10.1016/S0889-5406(98)70232-X

191. Mills CM, McCulloch KJ (2000) Posttreatment changes after successful correction of Class II malocclusions with the twin block appliance. American Journal of Orthodontics & Dentofacial Orthopedics 118(1): 24–33

192. Minase RA, Bhad WA, Doshi UH (2019) Effectiveness of reverse twin block with lip pads-RME and face mask with RME in the early treatment of class III malocclusion. Progress in Orthodontics 20(1): 14. doi: 10.1186/s40510-019-0266-0

193. Ming Y, Hu Y, Li Y et al. (2018) Effects of maxillary protraction appliances on airway dimensions in growing class III maxillary retrognathic patients: A systematic review and meta-analysis. [Review]. International Journal of Pediatric Otorhinolaryngology 105: 138–145. doi: 10.1016/j.ijporl.2017.12.013

194. Mutinelli S, Cozzani M (2015) Rapid maxillary expansion in early-mixed dentition: effectiveness of increasing arch dimension with anchorage on deciduous teeth. European Journal of Paediatric Dentistry 16(2): 115–122

195. Mutinelli S, Manfredi M, Guiducci A et al. (2015) Anchorage onto deciduous teeth: effectiveness of early rapid maxillary expansion in increasing dental arch dimension and improving anterior crowding. Progress in Orthodontics 16: 22. doi: 10.1186/s40510-015-0093-x

196. Muto T, Yamazaki A, Takeda S (2008) A cephalometric evaluation of the pharyngeal airway space in patients with mandibular retrognathia and prognathia, and normal subjects. Int. J. Oral Maxillofac. Surg. 37(3): 228–231. doi: 10.1016/j.ijom.2007.06.020

197. Nalbantgil D, Arun T, Sayinsu K et al. (2005) Skeletal, dental and soft-tissue changes induced by the Jasper Jumper appliance in late adolescence. Angle Orthodontist 75(3): 426–436

198. Ngan P, Yiu C, Hu A et al. (1998) Cephalometric and occlusal changes following maxillary expansion and protraction. European Journal of Orthodontics 20(3): 237–254

199. Nguyen QV, Bezemer PD, Habets L et al. (1999) A systematic review of the relationship between overjet size and traumatic dental injuries. Eur. J. Orthodont. 21(5): 503–515. doi: 10.1093/ejo/21.5.503

200. Nienkemper M, Wilmes B, Franchi L et al. (2015) Effectiveness of maxillary protraction using a hybrid hyrax-facemask combination: a controlled clinical study. Angle Orthodontist 85(5): 764–770. doi: 10.2319/071614-497.1

201. Nucera R, Lo,Giudice,A, Rustico L et al. (2016) Effectiveness of orthodontic treatment with functional appliances on maxillary growth in the short term: A systematic review

and meta-analysis. [Review]. American Journal of Orthodontics & Dentofacial Orthopedics 149(5): 600-611.e3. doi: 10.1016/j.ajodo.2015.09.030

202. O'Brien K, Macfarlane T, Wright J et al. (2009) Early treatment for Class II malocclusion and perceived improvements in facial profile. American Journal of Orthodontics & Dentofacial Orthopedics 135(5): 580–585. doi: 10.1016/j.ajodo.2008.02.020

203. O'Brien K, Wright J, Conboy F et al. (2003) Effectiveness of early orthodontic treatment with the Twin-block appliance: a multicenter, randomized, controlled trial. Part 1: Dental and skeletal effects. American Journal of Orthodontics & Dentofacial Orthopedics 124(3): 234-43; quiz 339

204. O'Brien K, Wright J, Conboy F et al. (2003) Effectiveness of early orthodontic treatment with the Twin-block appliance: a multicenter, randomized, controlled trial. Part 2: Psychosocial effects. American Journal of Orthodontics & Dentofacial Orthopedics 124(5): 488-94; discussion 494-5

205. Oh H, Baumrind S, Korn EL et al. (2017) A retrospective study of Class II mixed-dentition treatment. Angle Orthodontist 87(1): 56–67. doi: 10.2319/012616-72.1

206. Oz U, Orhan K, Rubenduz M (2013) Two-dimensional lateral cephalometric evaluation of varying types of Class II subgroups on posterior airway space in postadolescent girls: a pilot study. Journal of Orofacial Orthopedics 74(1): 18–27. doi: 10.1007/s00056-012-0121-0

207. Oztoprak MO, Nalbantgil D, Uyanlar A et al. (2012) A cephalometric comparative study of class II correction with Sabbagh Universal Spring (SUS(2)) and Forsus FRD appliances. European journal of dentistry 6(3): 302–310

208. Palomares NB, Celeste RK, Oliveira BH et al. (2012) How does orthodontic treatment affect young adults' oral health-related quality of life? American Journal of Orthodontics & Dentofacial Orthopedics 141(6): 751–758. doi: 10.1016/j.ajodo.2012.01.015

209. Pancherz H (1979) Treatment of class II malocclusions by jumping the bite with the Herbst appliance. A cephalometric investigation. American Journal of Orthodontics 76(4): 423–442

210. Pancherz H (1982) The mechanism of Class II correction in Herbst appliance treatment. A cephalometric investigation. American Journal of Orthodontics 82(2): 104–113

211. Pangrazio MNK, Pangrazio-Kulbersh V, Berger JL et al. (2012) Treatment effects of the mandibular anterior repositioning appliance in patients with Class II skeletal malocclusions. Angle Orthod. 82(6): 971–977. doi: 10.2319/120511-748.1

212. Papadopoulos MA, Melkos AB, Athanasiou AE (2010) Noncompliance maxillary molar distalization with the first class appliance: a randomized controlled trial. American Journal of Orthodontics & Dentofacial Orthopedics 137(5): 586.e1-586.e13; discussion 586-7. doi: 10.1016/j.ajodo.2009.10.033

213. Papageorgiou SN, Kutschera E, Memmert S et al. (2017) Effectiveness of early orthopaedic treatment with headgear: a systematic review and meta-analysis. [Review]. European Journal of Orthodontics 39(2): 176–187. doi: 10.1093/ejo/cjw041

214. Pavoni C, Cretella,Lombardo,E, Lione R et al. (2017) Orthopaedic treatment effects of functional therapy on the sagittal pharyngeal dimensions in subjects with sleep-

disordered breathing and Class II malocclusion. Acta Otorhinolaryngologica Italica 37(6): 479–485. doi: 10.14639/0392-100X-1420

215. Pavoni C, Gazzani F, Franchi L et al. (2019) Soft tissue facial profile in Class III malocclusion: long-term post-pubertal effects produced by the Face Mask Protocol. European Journal of Orthodontics 019. doi: 10.1093/ejo/cjz003

216. Pavoni C, Lombardo EC, Franchi L et al. (2017) Treatment and post-treatment effects of functional therapy on the sagittal pharyngeal dimensions in Class II subjects. Int. J. Pediatr. Otorhinolaryngol. 101: 47–50. doi: 10.1016/j.ijporl.2017.07.032

217. Pavoni C, Lombardo EC, Lione R et al. (2018) Treatment timing for functional jaw orthopaedics followed by fixed appliances: a controlled long-term study. European Journal of Orthodontics 40(4): 430–436. doi: 10.1093/ejo/cjx078

218. Peres KG, Barros AJD, Anselmi L et al. (2008) Does malocclusion influence the adolescent's satisfaction with appearance? A cross-sectional study nested in a Brazilian birth cohort. Community Dentist. Oral Epidemiol. 36(2): 137–143. doi: 10.1111/j.1600-0528.2007.00382.x

219. Perillo L, Castaldo MI, Cannavale R et al. (2011) Evaluation of long-term effects in patients treated with Frankel-2 appliance. European Journal of Paediatric Dentistry 12(4): 261–266

220. Perillo L, Johnston,LE,Jr, Ferro A (1996) Permanence of skeletal changes after function regulator (FR-2) treatment of patients with retrusive Class II malocclusions. American Journal of Orthodontics & Dentofacial Orthopedics 109(2): 132–139

221. Perillo L, Vitale M, Masucci C et al. (2016) Comparisons of two protocols for the early treatment of Class III dentoskeletal disharmony. European Journal of Orthodontics 38(1): 51–56

222. Perinetti G, Primozic J, Franchi L et al. (2015) Treatment Effects of Removable Functional Appliances in Pre-Pubertal and Pubertal Class II Patients: A Systematic Review and Meta-Analysis of Controlled Studies. [Review]. PLoS ONE [Electronic Resource] 10(10): e0141198. doi: 10.1371/journal.pone.0141198

223. Petren S, Bondemark L (2008) Correction of unilateral posterior crossbite in the mixed dentition: a randomized controlled trial. American Journal of Orthodontics & Dentofacial Orthopedics 133(6): 790.e7-13. doi: 10.1016/j.ajodo.2007.11.021

224. Petti S (2015) Over two hundred million injuries to anterior teeth attributable to large overjet: a meta-analysis. Dental Traumatology 31(1): 1–8. doi: 10.1111/edt.12126

225. Petti S, Tarsitani G (1996) Traumatic injuries to anterior teeth in Italian schoolchildren: prevalence and risk factors. Endod. Dent. Traumatol. 12(6): 294–297. doi: 10.1111/j.1600-9657.1996.tb00530.x

226. Piassi E, Antunes LS, Antunes LA (2016) Orthodontic treatment reduces the impact on children and adolescents' oral health-related quality of life. [Review]. Indian Journal of Dental Research 27(2): 213–219. doi: 10.4103/0970-9290.183122

227. Piassi E, Antunes LS, Graça TCA et al. (2019) The Impact of Mixed Dentition Malocclusion on the Oral Health-Related Quality of Life for Children and Their

Families: A Case-Control Study. J. Clin. Pediatr. Dent. 43(3): 211–217. doi: 10.17796/1053-4625-43.3.12

228. Pirilä-Parkkinen K, Pirttiniemi P, Nieminen P et al. (2009) Dental arch morphology in children with sleep-disordered breathing. Eur. J. Orthodont. 31(2): 160–167. doi: 10.1093/ejo/cjn061

229. Pithon MM, Santos NL, Santos CR et al. (2016) Is alternate rapid maxillary expansion and constriction an effective protocol in the treatment of Class III malocclusion? A systematic review. Dental Press Journal of Orthodontics 21(6): 34–42. doi: 10.1590/2177-6709.21.6.034-042.oar

230. Pontes LF, Maia FA, Almeida MR et al. (2017) Mandibular Protraction Appliance Effects in Class II Malocclusion in Children, Adolescents and Young Adults. Brazilian Dental Journal 28(2): 225–233. doi: 10.1590/0103-6440201701032

231. Prado RF, Ramos-Jorge J, Marques LS et al. (2016) Prospective evaluation of the psychosocial impact of the first 6 months of orthodontic treatment with fixed appliance among young adults. Angle Orthodontist 86(4): 644–648. doi: 10.2319/063015-434.1

232. Primo-Miranda EF, Ramos-Jorge ML, Homem MA et al. (2019) Association between occlusal characteristics and the occurrence of dental trauma in preschool children: a case-control study. Dental Traumatology 35(2): 95–100. doi: 10.1111/edt.12457

233. Quintao C, Helena I, Brunharo VP et al. (2006) Soft tissue facial profile changes following functional appliance therapy. European Journal of Orthodontics 28(1): 35–41

234. Raveli TB, Raveli DB, Gandini LG et al. (2017) Dental skeletal effects of the metallic splinted Herbst appliance after growth spurt: a lateral oblique cephalometric assessment. Acta Odontologica Latinoamericana 30(2): 76–82

235. Ren Y, Steegman R, Dieters A et al. (2019) Bone-anchored maxillary protraction in patients with unilateral complete cleft lip and palate and Class III malocclusion. Clinical Oral Investigations 23(5): 2429–2441. doi: 10.1007/s00784-018-2627-3

236. Rey D, Angel D, Oberti G et al. (2008) Treatment and posttreatment effects of mandibular cervical headgear followed by fixed appliances in Class III malocclusion. American Journal of Orthodontics & Dentofacial Orthopedics 133(3): 371-8; quiz 476.e1. doi: 10.1016/j.ajodo.2006.04.043

237. Rizk S, Kulbersh VP, Al-Qawasmi R (2016) Changes in the oropharyngeal airway of Class II patients treated with the mandibular anterior repositioning appliance. Angle Orthodontist 86(6): 955–961

238. Rodrigues,de,Almeida,M, Castanha,Henriques,JF, Rodrigues,de,Almeida,R et al. (2002) Treatment effects produced by Frankel appliance in patients with class II, division 1 malocclusion. Angle Orthodontist 72(5): 418–425

239. Rodriguez,de,Guzman-Barrera,J, Saez,Martinez,C, Boronat-Catala M et al. (2017) Effectiveness of interceptive treatment of class III malocclusions with skeletal anchorage: A systematic review and meta-analysis. PLoS ONE [Electronic Resource] 12(3): e0173875. doi: 10.1371/journal.pone.0173875

240. Rongo R, D'Anto V, Bucci R et al. (2017) Skeletal and dental effects of Class III orthopaedic treatment: a systematic review and meta-analysis. [Review]. Journal of Oral Rehabilitation 44(7): 545–562. doi: 10.1111/joor.12495

241. Ruf S, Pancherz H (1999) Dentoskeletal effects and facial profile changes in young adults treated with the Herbst appliance. Angle Orthodontist 69(3): 239–246

242. Ryan V, Chris AM, Dischinger T et al. (2006) Treatment effects of the edgewise Herbst appliance: A cephalometric and tomographic investigation. Am. J. Orthod. Dentofac. Orthop. 130(5): 582–593. doi: 10.1016/j.ajodo.2005.01.030

243. Saikoski LZ, Cancado RH, Valarelli FP et al. (2014) Dentoskeletal effects of Class II malocclusion treatment with the Twin Block appliance in a Brazilian sample: a prospective study. Dental Press Journal of Orthodontics 19(1): 36–45

244. Sambataro S, Fastuca R, Oppermann NJ et al. (2017) Cephalometric changes in growing patients with increased vertical dimension treated with cervical headgear. Journal of Orofacial Orthopedics 78(4): 312–320. doi: 10.1007/s00056-017-0087-z

245. Santamaria-Villegas A, Manrique-Hernandez R, Alvarez-Varela E et al. (2017) Effect of removable functional appliances on mandibular length in patients with class II with retrognathism: systematic review and meta-analysis. [Review]. BMC Oral Health 17(1): 52. doi: 10.1186/s12903-017-0339-8

246. Sauer C, Schlüter B, Hinz R et al. (2012) Childhood obstructive sleep apnea syndrome: an interdisciplinary approach: a prospective epidemiological study of 4,318 five-and-a-half-year-old children. Journal of Orofacial Orthopedics 73(5): 342–358. doi: 10.1007/s00056-012-0096-x

247. Schatz J-P, Hakeberg M, Ostini E et al. (2013) Prevalence of traumatic injuries to permanent dentition and its association with overjet in a Swiss child population. Dental Traumatology 29(2): 110–114. doi: 10.1111/j.1600-9657.2012.01150.x

248. Schiavon,Gandini,MR, Gandini,LG,Jr, Da,Rosa,Martins,JC et al. (2001) Effects of cervical headgear and edgewise appliances on growing patients. American Journal of Orthodontics & Dentofacial Orthopedics 119(5): 531-8; discussion 538-9

249. Schulz S, Koos B, Duske K et al. (2016) Skeletal effects in Angle Class II/1 patients treated with the functional regulator type II : Cephalometric and tensor analysis. Journal of Orofacial Orthopedics 77(6): 420–431

250. Seehra J, Fleming PS, Mandall N et al. (2012) A comparison of two different techniques for early correction of Class III malocclusion. Angle Orthodontist 82(1): 96–101. doi: 10.2319/032011-197.1

251. Seehra J, Fleming PS, Newton T et al. (2011) Bullying in orthodontic patients and its relationship to malocclusion,self-esteem and oral health-related quality of life. Journal of Orthodontics 38(4): 247-56; quiz 294. doi: 10.1179/14653121141641

252. Seehra J, Newton JT, DiBiase AT (2013) Interceptive orthodontic treatment in bullied adolescents and its impact on self-esteem and oral-health-related quality of life. European Journal of Orthodontics 35(5): 615–621. doi: 10.1093/ejo/cjs051

253. Sepanian VF, Sonnesen L (2018) Incisor root resorption in class II division 2 patients in relation to orthodontic treatment. Eur. J. Orthodont. 40(3): 337–342. doi: 10.1093/ejo/cjx086

254. Servello DF, Fallis DW, Alvetro L (2015) Analysis of Class II patients, successfully treated with the straight-wire and Forsus appliances, based on cervical vertebral maturation status. Angle Orthodontist 85(1): 80–86. doi: 10.2319/102513-780.1

255. Sideri S, Papageorgiou SN, Eliades T (2017) Are orthodontic systematic reviews registered a priori in PROSPERO? Journal of Orthodontics 44(4): 249–255. doi: 10.1080/14653125.2017.1370773

256. Sierwald I, John MT, Schierz O et al. (2015) Association of overjet and overbite with esthetic impairments of oral health-related quality of life. J Orofac Orthop 76(5): 405–420. doi: 10.1007/s00056-015-0300-x

257. Siluvai S, Kshetrimayum N, Reddy CVK et al. (2015) Malocclusion and related quality of life among 13- to 19-year-old students in Mysore City - a cross-sectional study. Oral Health Prev. Dent. 13(2): 135–141. doi: 10.3290/j.ohpd.a32339

258. Silvestrini-Biavati A, Alberti G, Silvestrini,Biavati,F et al. (2012) Early functional treatment in Class II division 1 subjects with mandibular retrognathia using Frankel II appliance. A prospective controlled study. European Journal of Paediatric Dentistry 13(4): 301–306

259. Silvola A-S, Närhi L, Tolvanen M et al. (2020) Gender-specific associations of malocclusion traits with oral health-related quality of life in a Finnish adult population. Eur. J. Orthodont. 42(3): 242–249. doi: 10.1093/ejo/cjz026

260. So LL (1996) Effects of reverse headgear treatment on sagittal correction in girls born with unilateral complete cleft lip and cleft palate--skeletal and dental changes. American Journal of Orthodontics & Dentofacial Orthopedics 109(2): 140–147

261. Soares TRC, Magno MB, Jural LA et al. (2018) Risk factors for traumatic dental injuries in the Brazilian population: A critical review. Dental Traumatology 34(6): 445–454. doi: 10.1111/edt.12439

262. Sollenius O, Golez A, Primozic J et al. (2019) Three-dimensional evaluation of forced unilateral posterior crossbite correction in the mixed dentition: a randomized controlled trial. European Journal of Orthodontics 019. doi: 10.1093/ejo/cjz054

263. Sonnesen L, Svensson P (2008) Temporomandibular disorders and psychological status in adult patients with a deep bite. Eur. J. Orthodont. 30(6): 621–629. doi: 10.1093/ejo/cjn044

264. Spalj S, Tranesen KM, Birkeland K et al. (2017) Comparison of Activator-Headgear and Twin Block Treatment Approaches in Class II Division 1 Malocclusion. Biomed Res. Int. doi: 10.1155/2017/4861924

265. Suzuki J, Shimazaki K, Koike S et al. (2018) Gastric emptying rate before and after orthodontic treatment examined with the $[^{13}C]$ breath test: A pilot study. American Journal of Orthodontics & Dentofacial Orthopedics 153(3): 347–354. doi: 10.1016/j.ajodo.2017.06.025

266. Tai K, Park JH, Mishima K et al. (2011) 3-Dimensional cone-beam computed tomography analysis of transverse changes with Schwarz appliances on both jaws. Angle Orthodontist 81(4): 670–677. doi: 10.2319/110910-655.1

267. Takemoto Y, Saitoh I, Iwasaki T et al. (2011) Pharyngeal airway in children with prognathism and normal occlusion. Angle Orthodontist 81(1): 75–80. doi: 10.2319/013010-65.1

268. Tepedino M, Della,Noce,MV, Ciavarella D et al. (2019) Soft-tissue changes after Class II malocclusion treatment using the Sander bite-jumping appliance: a retrospective study. Minerva Stomatologica 68(3): 118–125. doi: 10.23736/S0026-4970.19.04197-9

269. Thiruvenkatachari B, Harrison J, Worthington H et al. (2015) Early orthodontic treatment for Class II malocclusion reduces the chance of incisal trauma: Results of a Cochrane systematic review. [Review]. American Journal of Orthodontics & Dentofacial Orthopedics 148(1): 47–59. doi: 10.1016/j.ajodo.2015.01.030

270. Thiruvenkatachari B, Harrison JE, Worthington HV et al. (2013) Orthodontic treatment for prominent upper front teeth (Class II malocclusion) in children. [Review]. Cochrane Database of Systematic Reviews 013(11): CD003452. doi: 10.1002/14651858.CD003452.pub3

271. Tindlund RS, Rygh P (1993) Maxillary protraction: different effects on facial morphology in unilateral and bilateral cleft lip and palate patients. Cleft Palate Craniofacial Journal 30(2): 208–221

272. Toffol LD, Pavoni C, Baccetti T et al. (2008) Orthopedic treatment outcomes in Class III malocclusion. A systematic review. [Review] [40 refs]. Angle Orthodontist 78(3): 561–573. doi: 10.2319/030207-108.1

273. Tollaro I, Baccetti T, Franchi L (1996) Craniofacial changes induced by early functional treatment of Class III malocclusion. American Journal of Orthodontics & Dentofacial Orthopedics 109(3): 310–318

274. Toro A, Buschang PH, Throckmorton G et al. (2006) Masticatory performance in children and adolescents with Class I and II malocclusions. Eur. J. Orthodont. 28(2): 112–119. doi: 10.1093/ejo/cji080

275. Torre H, Alarcon J-A (2012) Changes in nasal air flow and school grades after rapid maxillary expansion in oral breathing children. Med. Oral Patol. Oral Cir. Bucal 17(5): E865-E870. doi: 10.4317/medoral.17810

276. Tortop T, Kaygisiz E, Gencer D et al. (2014) Modified tandem traction bow appliance compared with facemask therapy in treating Class III malocclusions. Angle Orthodontist 84(4): 642–648. doi: 10.2319/080513-584.1

277. Toth LR, McNamara,JA,Jr (1999) Treatment effects produced by the twin-block appliance and the FR-2 appliance of Frankel compared with an untreated Class II sample. American Journal of Orthodontics & Dentofacial Orthopedics 116(6): 597–609

278. Trankmann J, Lisson JA, Treutlein C (2001) Different orthodontic treatment effects in Angle Class III patients. Journal of Orofacial Orthopedics 62(5): 327–336

279. Trawitzki LV, Dantas RO, Mello-Filho FV et al. (2010) Masticatory muscle function three years after surgical correction of class III dentofacial deformity. International

Journal of Oral & Maxillofacial Surgery 39(9): 853–856. doi: 10.1016/j.ijom.2009.03.006

280. Trenouth MJ (2000) Cephalometric evaluation of the Twin-block appliance in the treatment of Class II Division 1 malocclusion with matched normative growth data. American Journal of Orthodontics & Dentofacial Orthopedics 117(1): 54–59

281. Trenouth MJ (2002) Proportional changes in cephalometric distances during Twin Block appliance therapy. European Journal of Orthodontics 24(5): 485–491

282. Trenouth MJ (2006) Centroid analysis of twin-block appliance treatment for Class II Division 1 malocclusion. World Journal of Orthodontics 7(2): 159–164

283. Trenouth MJ, Mew JR, Gibbs WW (2001) A cephalometric evaluation of the Biobloc technique using matched normative data. Journal of Orofacial Orthopedics 62(6): 466–475

284. Tristão, Sylvia Karla P C, Magno MB, Pintor AVB et al. (2020) Is there a relationship between malocclusion and bullying? A systematic review. Progress in Orthodontics 21(1): 26. doi: 10.1186/s40510-020-00323-7

285. Tsiouli K, Topouzelis N, Papadopoulos MA et al. (2017) Perceived facial changes of Class II Division 1 patients with convex profiles after functional orthopedic treatment followed by fixed orthodontic appliances. Am. J. Orthod. Dentofac. Orthop. 152(1): 80–91. doi: 10.1016/j.ajodo.2016.12.017

286. Tulloch JF, Phillips C, Koch G et al. (1997) The effect of early intervention on skeletal pattern in Class II malocclusion: a randomized clinical trial. American Journal of Orthodontics & Dentofacial Orthopedics 111(4): 391–400

287. Tulloch JF, Proffit WR, Phillips C (2004) Outcomes in a 2-phase randomized clinical trial of early Class II treatment. American Journal of Orthodontics & Dentofacial Orthopedics 125(6): 657–667

288. Tumer N, Gultan AS (1999) Comparison of the effects of monoblock and twin-block appliances on the skeletal and dentoalveolar structures. American Journal of Orthodontics & Dentofacial Orthopedics 116(4): 460–468

289. Tuncer BB, Kaygisiz E, Tuncer C et al. (2009) Pharyngeal airway dimensions after chin cup treatment in Class III malocclusion subjects. J. Oral Rehabil. 36(2): 110–117. doi: 10.1111/j.1365-2842.2008.01910.x

290. Turkkahraman H, Sayin MO (2006) Effects of activator and activator headgear treatment: comparison with untreated Class II subjects. Eur. J. Orthodont. 28(1): 27–34. doi: 10.1093/ejo/cji062

291. Ucem TT, Ucuncu N, Yuksel S (2004) Comparison of double-plate appliance and facemask therapy in treating Class III malocclusions. Am. J. Orthod. Dentofac. Orthop. 126(6): 672–679. doi: 10.1016/j.ajodo.2003.09.035

292. Ulger G, Arun T, Sayinsu K et al. (2006) The role of cervical headgear and lower utility arch in the control of the vertical dimension. American Journal of Orthodontics & Dentofacial Orthopedics 130(4): 492–501

293. Ulusoy C, Canigur,Bavbek,N, Tuncer BB et al. (2014) Evaluation of airway dimensions and changes in hyoid bone position following class II functional therapy with activator. Acta Odontologica Scandinavica 72(8): 917–925. doi: 10.3109/00016357.2014.923109

294. Usumez S, Uysal T, Sari Z et al. (2004) The effects of early preorthodontic trainer treatment on Class II, division 1 patients. Angle Orthodontist 74(5): 605–609

295. van Lierde KM, Luyten A, D'haeseleer E et al. (2015) Articulation and oromyofunctional behavior in children seeking orthodontic treatment. Oral Dis. 21(4): 483–492. doi: 10.1111/odi.12307

296. Vanlaecken R, Martin CA, Dischinger T et al. (2006) Treatment effects of the edgewise Herbst appliance: a cephalometric and tomographic investigation. American Journal of Orthodontics & Dentofacial Orthopedics 130(5): 582–593

297. Varlik SK, Iscan HN (2008) The effects of cervical headgear with an expanded inner bow in the permanent dentition. European Journal of Orthodontics 30(4): 425–430. doi: 10.1093/ejo/cjn016

298. Vázquez-Casas I, Sans-Capdevila O, Moncunill-Mira J et al. (2020) Prevalence of sleep-related breathing disorders in children with malocclusion. Journal of Clinical & Experimental Dentistry 12(6): e555-e560. doi: 10.4317/jced.56855

299. Vilanova L, Henriques JF, Janson G et al. (2018) Class II malocclusion treatment effects with Jones Jig and Distal Jet followed by fixed appliances. Angle Orthodontist 88(1): 10–19

300. Watkinson S, Harrison JE, Furness S et al. (2013) Orthodontic treatment for prominent lower front teeth (Class III malocclusion) in children. [Review]. Cochrane Database of Systematic Reviews 013(9): CD003451. doi: 10.1002/14651858.CD003451.pub2

301. Wendl B, Muchitsch AP, Winsauer H et al. (2017) Retrospective 25-year follow-up of treatment outcomes in angle Class III patients : Early versus late treatment. Journal of Orofacial Orthopedics 78(3): 201–210. doi: 10.1007/s00056-016-0076-7

302. Westwood PV, McNamara JA, Baccetti T et al. (2003) Long-term effects of Class III treatment with rapid maxillary expansion and facemask therapy followed by fixed appliances. Am. J. Orthod. Dentofac. Orthop. 123(3): 306–320. doi: 10.1067/mod.2003.44

303. Wieslander L (1975) Early or late cervical traction therapy of Class II malocclusion in the mixed dentition. American Journal of Orthodontics 67(4): 432–439

304. Woon SC, Thiruvenkatachari B (2017) Early orthodontic treatment for Class III malocclusion: A systematic review and meta-analysis. [Review]. American Journal of Orthodontics & Dentofacial Orthopedics 151(1): 28–52. doi: 10.1016/j.ajodo.2016.07.017

305. Xiang M, Hu B, Liu Y et al. (2017) Changes in airway dimensions following functional appliances in growing patients with skeletal class II malocclusion: A systematic review and meta-analysis. [Review]. International Journal of Pediatric Otorhinolaryngology 97: 170–180. doi: 10.1016/j.ijporl.2017.04.009

306. Yagci A, Uysal T (2010) Effects of conventional and modified facemask therapies on dentofacial structures. Korean J. Orthod. 40(6): 432–443. doi: 10.4041/kjod.2010.40.6.432

307. Yang X, Li C, Bai D et al. (2014) Treatment effectiveness of Frankel function regulator on the Class III malocclusion: a systematic review and meta-analysis. [Review]. American Journal of Orthodontics & Dentofacial Orthopedics 146(2): 143–154. doi: 10.1016/j.ajodo.2014.04.017

308. Yavuz I, Halicioglu K, Ceylan I (2009) Face mask therapy effects in two skeletal maturation groups of female subjects with skeletal Class III malocclusions. Angle Orthodontist 79(5): 842–848. doi: 10.2319/090308-462.1

309. Zhang Y, Jia H, Fu Z et al. (2018) Dentoskeletal effects of facemask therapy in skeletal Class III cleft patients with or without bone graft. American journal of orthodontics and dentofacial orthopedics 153(4): 542–549

310. Zhao N, Feng J, Hu Z et al. (2015) Effects of a novel magnetic orthopedic appliance (MOA-III) on the dentofacial complex in mild to moderate skeletal class III children. Head & Face Medicine 11: 34. doi: 10.1186/s13005-015-0092-7

311. Zheng D-H, Wang X-X, Su Y-R et al. (2015) Assessing changes in quality of life using the Oral Health Impact Profile (OHIP) in patients with different classifications of malocclusion during comprehensive orthodontic treatment. BMC Oral Health 15: 148. doi: 10.1186/s12903-015-0130-7

312. Zhou Y, Long H, Ye N et al. (2014) The effectiveness of non-surgical maxillary expansion: a meta-analysis. European Journal of Orthodontics 36(2): 233–242. doi: 10.1093/ejo/cjt044

313. Zhou Y, Wang Y, Wang X et al. (2014) The impact of orthodontic treatment on the quality of life a systematic review. BMC Oral Health 14: 66. doi: 10.1186/1472-6831-14-66

314. Zou B, Zhou Y, Lowe AA et al. (2015) Changes in anteroposterior position and inclination of the maxillary incisors after surgical-orthodontic treatment of skeletal class III malocclusions. J. Cranio-MaxilloFac. Surg. 43(10): 1986–1993. doi: 10.1016/j.jcms.2014.12.013

315. Zymperdikas VF, Koretsi V, Papageorgiou SN et al. (2016) Treatment effects of fixed functional appliances in patients with Class II malocclusion: a systematic review and meta-analysis. [Review]. European Journal of Orthodontics 38(2): 113–126. doi: 10.1093/ejo/cjv034
